# Elevation of master autophagy regulator *Tfeb* in osteoblast lineage cells increases bone mass and strength

**DOI:** 10.1172/jci.insight.191688

**Published:** 2025-07-29

**Authors:** Alicen James, James A. Hendrixson, Ilham Kadhim, Adriana Marques-Carvalho, Jacob Laster, Julie Crawford, Jeff Thostenson, Visanu Wanchai, Amy Y. Sato, Intawat Nookaew, Jinhu Xiong, Maria Almeida, Melda Onal

**Affiliations:** 1 Department of Physiology and Cell Biology;; 2 Division of Endocrinology and Metabolism, Department of Internal Medicine;; 3 Center for Musculoskeletal Disease Research (CMDR);; 4 Department of Biostatistics;; 5 Department of Biomedical Informatics; and; 6 Department of Orthopaedic Surgery, University of Arkansas for Medical Sciences (UAMS), Little Rock, Arkansas, USA.

**Keywords:** Bone biology, Cell biology, Autophagy, Osteoclast/osteoblast biology, Osteoporosis

## Abstract

Autophagy is a recycling pathway in which damaged proteins, protein aggregates, and organelles are delivered to lysosomes for degradation. Autophagy insufficiency is thought to contribute to osteoporosis. Accordingly, autophagy elimination from the osteoblast lineage reduces bone formation and bone mass. However, whether increasing autophagy would benefit bone health is unknown. Here, we increased expression of endogenous transcription factor EB gene (*Tfeb*) in osteoblast lineage cells in vivo via CRISPR activation (*Tfeb*^CRa^ mice). Elevated *Tfeb* stimulated autophagy and lysosomal biogenesis in osteoblasts. *Tfeb*^CRa^ mice displayed a robust increase in femoral and vertebral cortical thickness at 4.5 months of age. Increases in cortical thickness were due to increased periosteal bone formation. *Tfeb* elevation also increased femoral trabecular bone volume. These changes increased bone strength of *Tfeb*^CRa^ mice. Female *Tfeb*^CRa^ mice displayed a progressive increase in bone mass and at 12 months of age had high cortical thickness and trabecular bone volume. Increased vertebral trabecular bone volume was due to elevated bone formation. Osteoblastic cultures showed that *Tfeb* elevation increased proliferation and mineral deposition. Overall, these results demonstrate TFEB-driven stimulation of autophagy in osteoblast lineage cells is associated with increased bone formation and strength and may represent an effective approach to combat osteoporosis.

## Abstract 2

Autophagy is a cellular recycling mechanism that is essential for bone formation. A genetic maneuver that stimulates autophagy increases bone formation, bone mass, and strength.

## Introduction

Throughout life, bone is remodeled by osteoclasts, which resorb bone, and osteoblasts, which form bone (1). As osteoblasts form new bone, some become embedded in the bone matrix they produce and differentiate into osteocytes. Osteocytes orchestrate bone remodeling by secreting factors that control bone formation and resorption (2). When the balance between bone formation and resorption shifts in favor of bone resorption, bone mass is lost. The health and functionality of osteoblast lineage cells (osteoblast progenitors, osteoblasts, and osteocytes) are essential for bone formation (3). Therefore, insight into the pathways and mechanisms that control the production, survival, and function of osteoblast lineage cells may identify new therapeutic targets.

Macroautophagy/autophagy is a catabolic process in which cellular contents are engulfed by autophagosomes and delivered to lysosomes for degradation and recycling. Autophagy clears damaged organelles and protein aggregates and turns over cytosolic components. Deletion of essential components of autophagy, such as autophagy related 7 (*Atg7*), *Atg5*, or unc-51 like kinase 1 (*Ulk1*), using various Cre driver strains active in osteoblast lineage cells demonstrates that autophagy is essential for the accrual of bone mass (4–8). Most strikingly, deletion of *Atg7* from the entire osteoblast lineage using the Osx1-Cre transgene drastically decreases osteoblast number and bone formation, leading to low bone mass and a 50% fracture rate (5). Together, these studies demonstrate that autophagy is essential for the maintenance of osteoblast number and bone formation and suggest that insufficient autophagy levels may contribute to the decline in bone formation in various skeletal pathologies, such as age-related bone loss (9–11). However, whether stimulation of autophagy in osteoblast lineage cells could be beneficial for bone in physiological or pathological conditions remains unknown.

To address this question, we sought to stimulate autophagy in osteoblast lineage cells by increasing expression of transcription factor EB (*Tfeb*) — the major transcriptional regulator of genes involved in autophagy and lysosomal biogenesis (12). This approach is based on evidence that targeted overexpression of *Tfeb* in myotubes (13), neurons (14–16), oligodendrocytes (15), macrophages (17), cardiac myocytes (18), or chondrocytes (19) is sufficient to induce autophagy and alleviate dysfunction in murine models of Pompe disease, Parkinson disease, Alzheimer disease, multiple system atrophy, atherosclerosis, cardiac hypertrophy, and osteoarthritis, respectively. Herein, we used in vivo CRISPR activation to elevate endogenous *Tfeb* expression to stimulate autophagy in osteoblast lineage cells. This genetic maneuver greatly increased bone mass by promoting bone formation.

## Results

### Generation of sgRNA^Tfeb^ mice.

**Figure 1:**
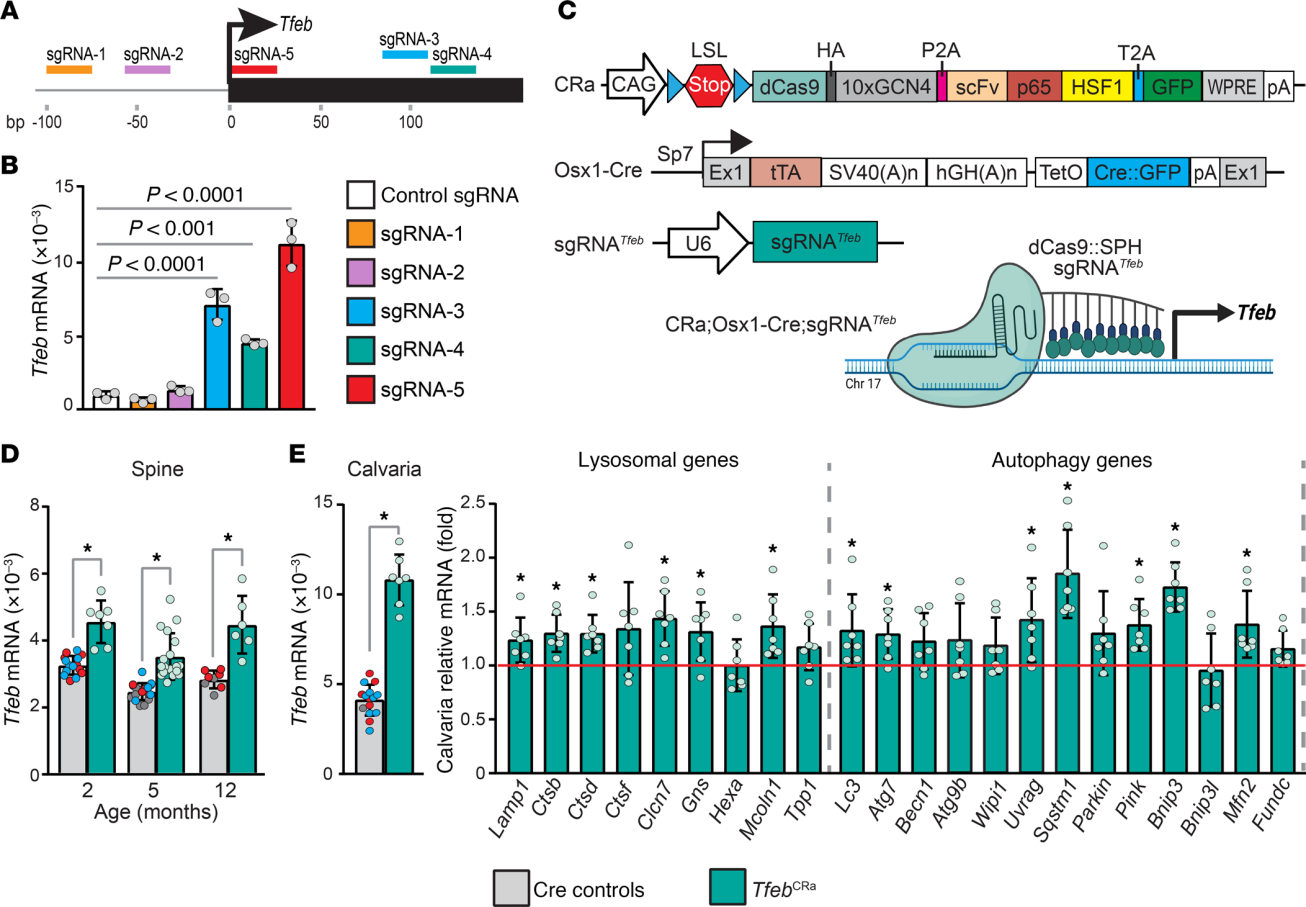
Elevation of *Tfeb* in osteoblast lineage cells via CRISPRa stimulates expression of genes involved in autophagy and lysosomal biogenesis. (**A**) Diagram depicting the chr17:47,736,921–47,737,191 of GRCm38/mm10 and showing positions of 5 sgRNAs in relation to the transcription start site (TSS) of *Tfeb* gene. To stimulate transcription of *Tfeb*, the SP-dCas9-VPR/sgRNA complex was targeted to within 150 bp of the TSS. (**B**) Gene expression analysis of osteoblastic UAMS-32 cells transfected with SP-dCas9-VPR and sgRNAs targeting *Tfeb*. *n* = 3 wells per group. *P* values are calculated by 1-way ANOVA followed by Dunnett’s multiple comparison test comparing each with the control group. (**C**) Transgene design of murine models used for CRISPRa. Specifically, CRISPR/dCas9-activator (CRa), Osx1-Cre, and sgRNA*^Tfeb^* mice were crossed to obtain triple transgenic CRa Osx1-Cre sgRNA*^Tfeb^* (*Tfeb*^CRa^) mice. (**D** and **E**) Comparison of Cre control mice, which include Osx1-Cre only (red dots), Osx1-Cre CRa (blue dots), and Osx1-Cre sgRNA*^Tfeb^* (gray dots), with *Tfeb*^CRa^ mice. (**D**) *Tfeb* mRNA levels were measured in lumbar vertebrae 5 (spine) of *Tfeb*^CRa^ mice and their littermate Cre controls by quantitative real-time PCR (qRT-PCR) at 2, 5, or 12 months of age. (**E**) mRNA levels of *Tfeb* and genes involved in lysosomal biogenesis and autophagy were measured in calvaria of 2-month-old *Tfeb*^CRa^ mice and their Cre littermate controls via qRT-PCR. For all qRT-PCR analyses, mRNA levels were normalized to mouse *Actb*. Lysosome- and autophagy-related gene expression is represented as fold change over Cre controls. *Lamp1*, lysosomal-associated membrane protein 1. *n* = 6–18 mice per group. Bars indicate mean ± SD. *P < 0.05 as calculated by unpaired *t* test for equal or unequal variance.

To increase *Tfeb* expression in vivo, we used CRISPR activation (CRISPRa) (20–23). In this methodology, nuclease-deficient Cas9 (dead Cas9, dCas9), which cannot cut DNA, is fused to one or more transcriptional activator domains (dCas9:activator). To increase transcription of target genes, the dCas9:activator fusion protein is directed to the proximity of the target gene’s transcriptional start site (TSS) via the use of an sgRNA that is complementary to that region. The level of transcriptional stimulation varies based on where the dCas9:activator is directed to relative to the TSS of the targeted gene. To identify sgRNAs that facilitate elevated expression of *Tfeb*, we designed 5 sgRNAs targeting the 300 bp region centered on the *Tfeb* TSS with minimal off-target potential (Figure 1A). We next tested their activity in vitro using an osteoblast cell line that expresses dCas9:activator (SP-dCas9-VPR) and identified 3 sgRNAs that elevated *Tfeb* expression 4- to 11-fold in culture (Figure 1B). Sustained high-level overexpression of genes can have detrimental effects. For example, in a previously described murine model, sustained high-level overexpression of *Tfeb* (8-fold compared with controls) in the heart had detrimental effects (24). To avoid potential complications because of high-level overexpression, for the generation of our mouse model, we selected *Tfeb*_sgRNA_4, which stimulates *Tfeb* expression moderately (Figure 1B).

To obtain ubiquitous and consistent expression of the selected sgRNA for CRISPRa, we inserted an expression cassette encoding it into a safe harbor locus. In previous studies, we successfully used this approach to express an sgRNA for in vivo CRISPR interference (25). Similarly, herein we introduced a cassette expressing *Tfeb*_sgRNA_4 into the murine *Rosa26* locus, producing sgRNA*^Tfeb^* mice. As expected, hemizygous or homozygous sgRNA*^Tfeb^* mice were born at expected Mendelian ratios and were grossly indistinguishable from wild-type mice.

### Tfeb elevation in the osteoblast lineage stimulates autophagy and lysosomal biogenesis.

To increase endogenous *Tfeb* in osteoblast lineage cells in vivo, we crossed sgRNA*^Tfeb^* mice with CRa (22) and Osx1-Cre mice (26) (Figure 1C). CRa transgenic mice contain a floxed stop cassette that prevents expression of dCas9:activator (dCas9:SunTag-p65-HSF1 or dCas9:SPH) until activated by a Cre driver strain (22). In triple transgenic CRa Osx1-Cre sgRNA*^Tfeb^* mice, from here on referred to as *Tfeb*^CRa^ mice, dCas9:SPH is expressed only in cells targeted by Osx1-Cre. *Tfeb*^CRa^ mice showed a ~1.4-fold increase in *Tfeb* mRNA in spine compared with controls (Figure 1D). Next, we examined the expression of lysosomal and autophagy-related genes whose expression is controlled by TFEB in multiple cell types (27–31). A 2.7-fold increase in *Tfeb* mRNA was also seen in calvariae of *Tfeb*^CRa^ mice, and this was associated with increased expression of several genes involved in lysosomal biogenesis and autophagy (Figure 1E). It was previously shown that TFEB can induce autophagy of mitochondria (mitophagy) in part by increasing expression of mitophagy receptors (30). Therefore, we also measured expression of mitophagy receptors, which are thought to be important for mesenchymal bone cells (32–36), and showed that TFEB elevated expression of many of these genes (Figure 1E), suggesting that mitophagy may be elevated in *Tfeb*^CRa^ osteoblasts. In support of this, we observed a reduction in mitochondrial mass of *Tfeb*^CRa^ osteoblasts (Supplemental Figure 1; supplemental material available online with this article; https://doi.org/10.1172/jci.insight.191688DS1).

**Figure 2:**
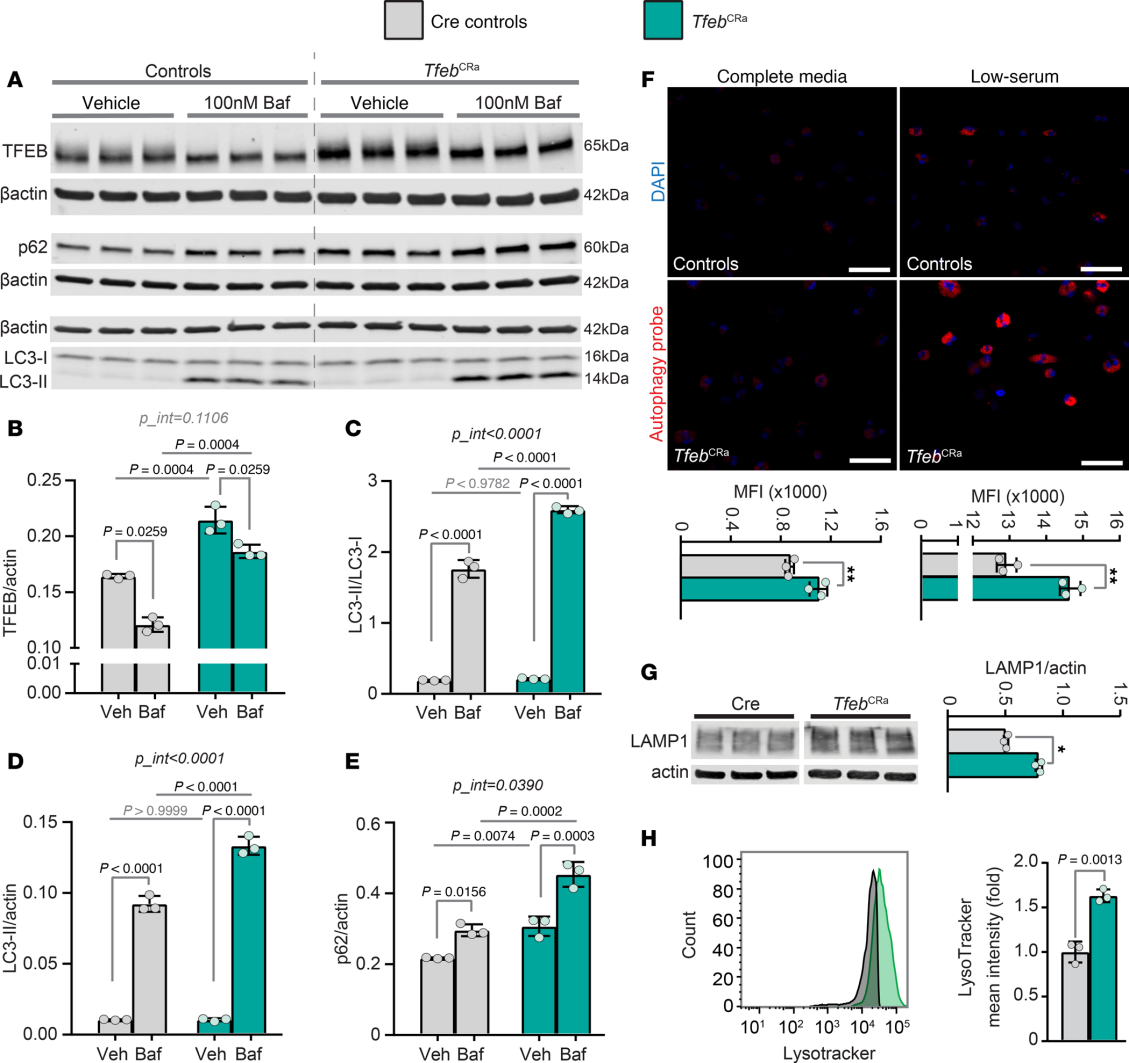
Elevation of TFEB in osteoblasts induces autophagy and lysosomal biogenesis. Bone marrow cells were isolated from femurs and tibias of 5-month-old female *Tfeb*^CRa^ mice and littermate Cre controls and differentiated into osteoblasts by culturing with osteogenic media for 21 days. (**A**–**E**) Osteoblasts were treated with vehicle (PBS) or 100 nM bafilomycin for 6 hours. (**A**) Western blot analysis was performed to measure and quantify TFEB (**B**), LC3 (**C** and **D**), p62 (**E**), and actin levels. (**F**) Prior to staining with a cell-permeable Autophagy Probe, media were replaced with complete (10% FBS) or low-serum (2% FBS) media for 2 hours. Images were acquired via confocal microscopy, and mean fluorescence intensity was measured by flow cytometry. Scale bar represents 50 μm. (**G**) Western blot analysis was performed to measure LAMP1 and actin levels. Samples were run on the same gel but were noncontiguous. (**H**) LysoTracker mean intensity was measured by flow cytometry. *n* = 3 wells per group. For all protein quantification, protein levels were normalized to actin levels. Bars indicate mean ± SD. Indicated *P* values were calculated by 2-way ANOVA followed by Tukey’s post hoc analysis (**B**–**E**) or unpaired *t* test (**F**–**H**). For **B**, 2-way ANOVA did not have normal residuals, so rank transformation was performed.

Next, we examined whether elevation of *Tfeb* stimulates autophagy in osteoblast lineage cells. To this end, we cultured cells isolated from femurs of *Tfeb*^CRa^ mice and littermate controls in osteogenic differentiation medium for 3 weeks, then treated these osteoblasts with bafilomycin (Baf) or vehicle. Western blot analysis verified that *Tfeb*^CRa^ osteoblasts had higher TFEB levels than Cre controls (Figure 2, A and B). Consistent with previous studies (37), inhibition of lysosomal function (Baf treatment) caused a shift in TFEB mobility (Figure 2A). This shift in TFEB mobility is likely because inhibition of lysosomal function decreases mTOR-dependent phosphorylation of TFEB (37). Next, we measured autophagy levels by examining LC3-I to -II conversion, LC3-II flux, and p62 flux (Figure 2, A–E). Autophagosome formation indicated by LC3-I to -II conversion was increased in *Tfeb*^CRa^ osteoblasts compared with Cre controls (Figure 2, A and C). Comparison of LC3-II and p62 degradation by 2-way ANOVA interaction analysis (*p*_int < 0.05) revealed higher degradation of these proteins in *Tfeb*^CRa^ osteoblasts compared with controls (Figure 2, A, D, and E), indicating higher autophagic flux in *Tfeb*^CRa^ osteoblasts. Independent measurement of autophagy using a cell-permeable fluorescent Autophagy Probe verified that autophagy is indeed higher in *Tfeb*^CRa^ osteoblasts compared with Cre controls when cultured in complete (10% FBS) or low-serum (2% FBS) media (Figure 2F). As indicated by LAMP1 protein levels and LysoTracker staining (Figure 2, G and H), TFEB elevation in osteoblasts also increased lysosomal biogenesis. Taken together, these results demonstrate that the elevation of endogenous *Tfeb* in osteoblast lineage cells is sufficient to stimulate autophagy and lysosomal biogenesis.

### Elevation of Tfeb in the osteoblast lineage increases bone mass and strength.

**Figure 3:**
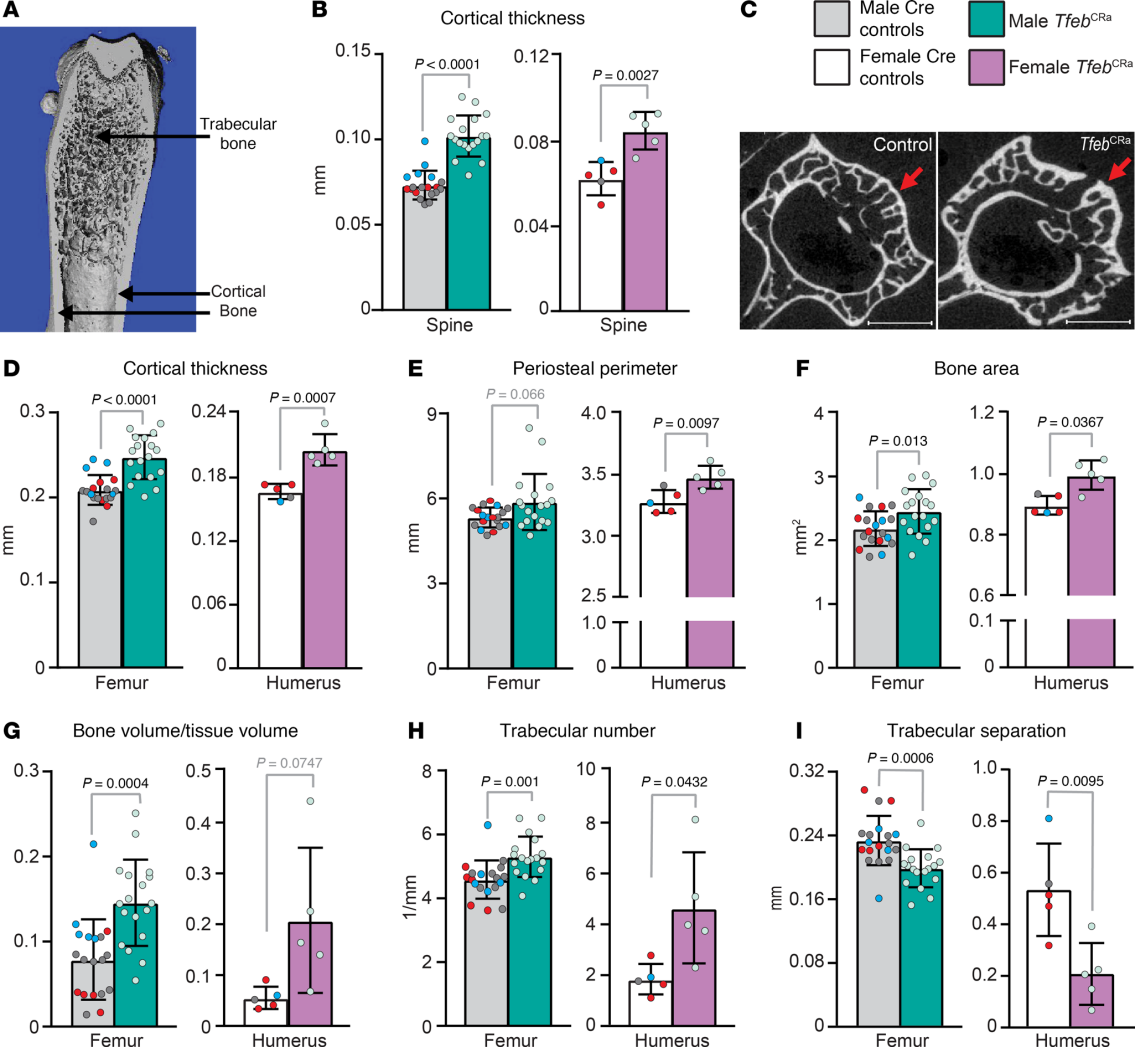
*Tfeb* elevation in the osteoblast lineage increases bone mass. (**A**) Representative μCT image of a mouse femur, indicating trabecular and cortical bone compartments. (**B**–**I**) μCT analysis performed on spine (lumbar vertebrae 4, L4) and long bones (male femurs and female humerus) from 4.5- to 5-month-old male and female *Tfeb*^CRa^ (Osx1-Cre CRa sgRNA*^Tfeb^*) and Cre control (Osx1-Cre only [red dots], Osx1-Cre CRa [blue dots], Osx1-Cre sgRNA*^Tfeb^* [gray dots]) mice. (**B**) Cortical thickness (Ct.Th) was measured in L4. (**C**) Representative L4 vertebrae images of *Tfeb*^CRa^ and control mice. Arrow indicates cortical bone. Scale bars represent 1 mm. (**D**–**F**) μCT analysis of Ct.Th (**D**), periosteal perimeter (Ps.Pm) (**E**), and bone area (**F**) measured at the long bone midshaft. (**G**–**I**) Trabecular bone volume over tissue volume (BV/TV) (**G**), trabecular number (Tb.N) (**H**), and trabecular separation (Tb.Sp) (**I**) were measured in the long bone distal metaphysis. Male, *n* = 19 control and *n* = 18 *Tfeb*^CRa^ mice; female, *n* = 5 mice per group. Bars indicate mean ± SD. Indicated *P* values were calculated by either unpaired *t* test for equal or unequal variance (**G**-female, **H**-female) or rank sum test (if data are not normally distributed, **B**-male, **E**-male, **F**-female, **G**-male).

To assess the skeletal impact of *Tfeb* elevation in osteoblast lineage cells, we performed detailed skeletal phenotyping of mice at 4.5 to 5 months of age. At this age bone growth is completed and peak bone mass is attained. We first used microCT analysis to examine the architectural changes in cortical and trabecular bone compartments. Cortical bone is the dense outer layer that encapsulates bone marrow and trabecular bone like a shell (Figure 3A). Trabecular bone is the inner bone compartment (lattice-like appearance) that is surrounded by bone marrow (Figure 3A). Cortical and trabecular bone compartments differ in their formation, maintenance, and response to mechanical or chemical stimuli (38, 39). As different skeletal sites have different ratios of cortical and trabecular bone (38), we examined changes in these 2 bone compartments in both the appendicular (represented by long bones) and axial (represented by spine) skeleton.

Analysis of male and female *Tfeb*^CRa^ mice revealed higher cortical thickness in both the spine and long bones (Figure 3, B–D). The change in cortical thickness in long bones was due to increased periosteal perimeter (outer bone surface) and bone area (Figure 3, E and F), while no changes were detected in the endosteal perimeter (inner bone surface) (Supplemental Figure 2A). In other words, elevation of *Tfeb* in Osx1-Cre–targeted cells led to widening of the bones. Regarding the trabecular compartment, *Tfeb* elevation in the osteoblast lineage increased the volume of trabecular bone in long bones (Figure 3G). This increase was due to presence of more trabeculae that were closer to one another (Figure 3, H and I). *Tfeb* elevation also caused a modest increase in trabecular thickness in spine (Supplemental Figure 2, B and C). These results demonstrate that elevation of *Tfeb* in osteoblast lineage cells is beneficial for both trabecular and cortical bone compartments in young adult male and female mice.

**Figure 4:**
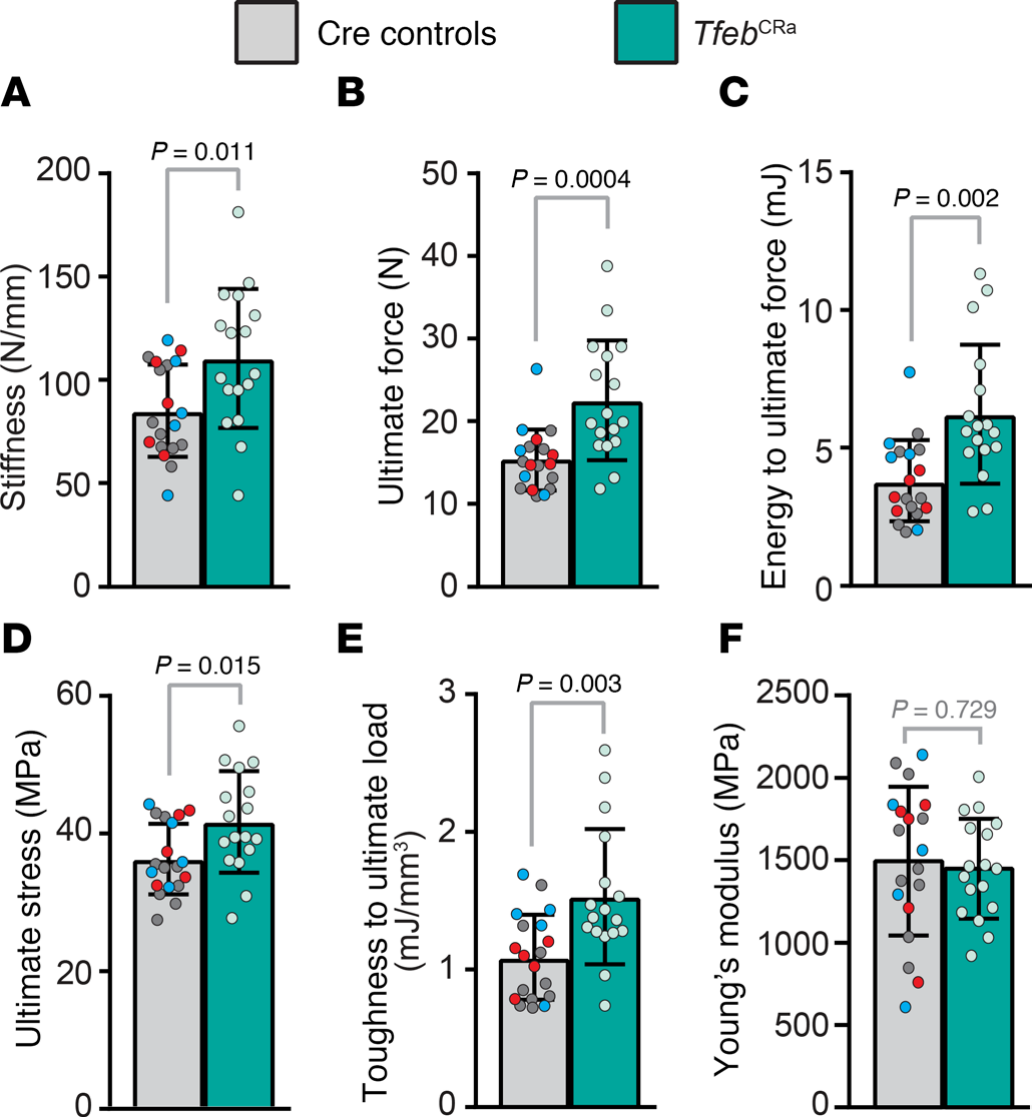
*Tfeb* elevation in the osteoblast lineage increases femoral strength. Femoral 3-point bending biomechanical testing was performed on 4.5-month-old male *Tfeb*^CRa^ mice and their littermate Cre controls (Osx1-Cre only [red dots], Osx1-Cre CRa [blue dots], Osx1-Cre sgRNA*^Tfeb^* [gray dots]) to assess extrinsic and intrinsic properties of bone. (**A**–**C**) Extrinsic properties include stiffness (**A**), ultimate force (**B**), and energy to ultimate force (**C**). (**D**–**F**) Intrinsic properties include ultimate stress (**D**), toughness to ultimate load (**E**), and Young’s modulus (**F**). *n* = 19 control and *n* = 17 *Tfeb*^CRa^ mice (please see Methods). Bars indicate mean ± SD. Indicated *P* values were calculated by unpaired *t* test for equal or unequal variance (**C**) or rank sum test (if data are not normally distributed, **B**).

Next, we examined whether these architectural changes improved biomechanical properties of *Tfeb*^CRa^ bones by performing 3-point bending testing of femurs. This analysis revealed that both extrinsic/structural (Figure 4, A–C) and intrinsic/material properties of the bone (Figure 4, D and E) were increased in *Tfeb*^CRa^ mice compared with controls. However, there were no differences in the Young’s modulus (Figure 4F), which is calculated as stiffness normalized by the tissue distribution and geometry. These results indicate that the gains in stiffness are due to beneficial changes in bone geometry and microarchitecture, as opposed to alterations in tissue properties (such as degree of mineralization or collagen cross-linking). In other words, *Tfeb* elevation in the osteoblast lineage increases the amount of bone, which is of normal quality, resulting in stronger bones.

### Elevation of Tfeb in the osteoblast lineage increases bone formation.

**Figure 5:**
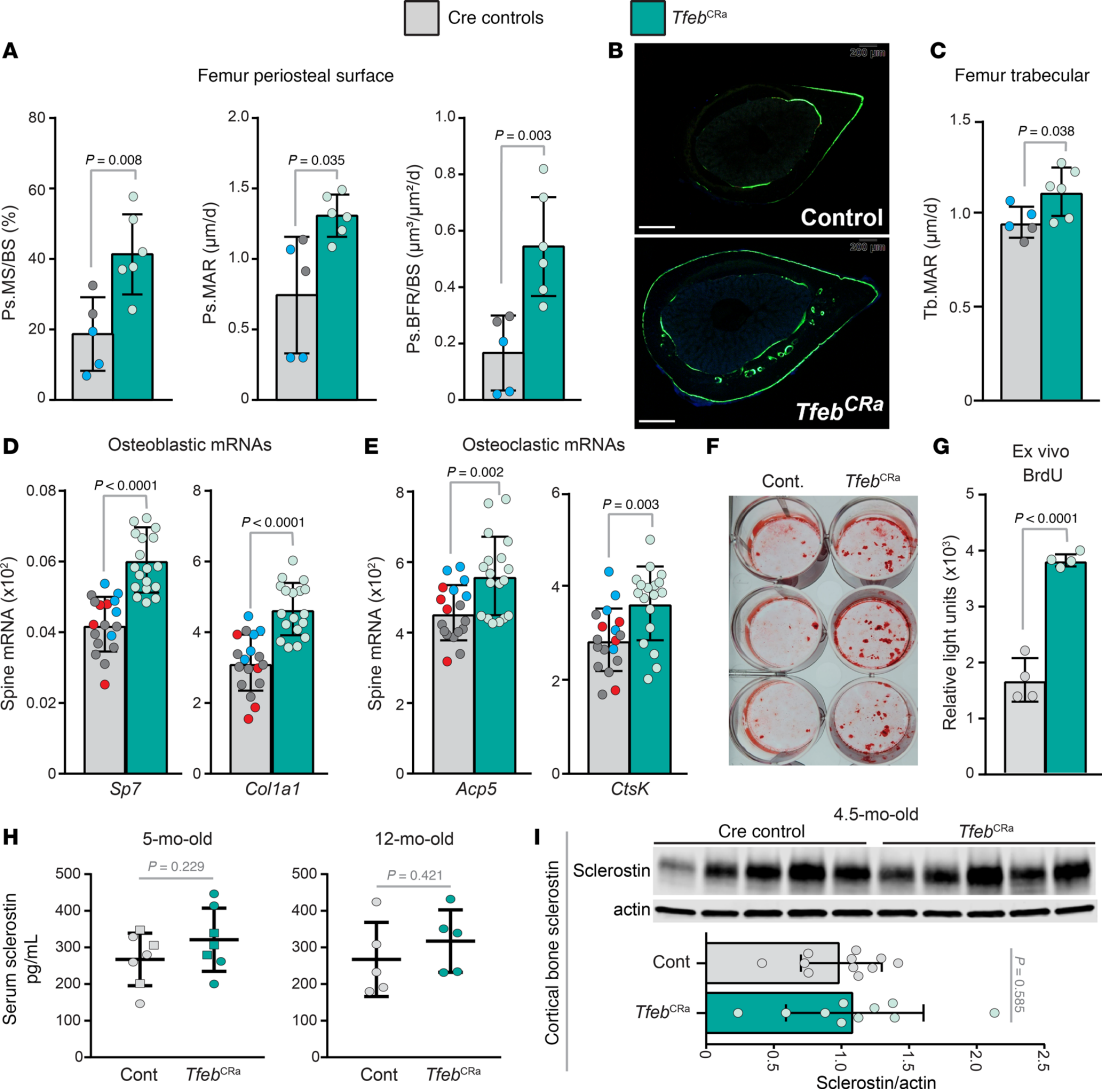
*Tfeb* elevation in the osteoblast lineage increases bone formation. (**A**–**E**) Skeletal phenotype analysis of 4.5-month-old male *Tfeb*^CRa^ mice and their littermate Cre controls (Osx1-Cre only [red dots], Osx1-Cre CRa [blue dots], Osx1-Cre sgRNA*^Tfeb^* [gray dots]). (**A**–**C**) Dynamic histomorphometry was performed on femurs. *n* = 5–6 mice per group. (**A**) Quantification of mineralizing surface per bone surface (MS/BS), mineral apposition rate (MAR), and bone formation rate per bone surface (BFR/BS) at the periosteal surface. (**B**) Representative histological cross section showing calcein labeling at the femoral diaphysis (GFP filter). Scale bars represent 400 μm. (**C**) Trabecular MAR (Tb. MAR). (**D** and **E**) *Sp7* and *Col1a1* (**D**) and *Acp5* and *Ctsk* (**E**) mRNA levels were measured in L5 by qRT-PCR and normalized to mouse *Actb*. *n* = 18 mice per group (please see Methods). (**F** and **G**) Bone marrow cells were isolated from femurs and tibias of 5-month-old female mice and differentiated into osteoblasts using osteogenic media. (**F**) Alizarin red stain of mineral apposition. (**G**) BrdU analysis of proliferating cells. *n* = 4 wells per group. (**H**) Circulating sclerostin levels of 5- and 12-month-old male (square) and female (circle) *Tfeb*^CRa^ and control mice were measured using ELISA. *n* = 5–7 mice per group. (**I**) Sclerostin and actin levels were measured in cortical bone (humeri shafts) of *Tfeb*^CRa^ and control mice via Western blot. Immunoblot of 5 mice per group is shown. Quantification was done with *n* = 10–11 mice per group. The bar graph indicates sclerostin levels normalized to actin. Bars indicate mean ± SD. Indicated *P* values were calculated by unpaired *t* test for equal or unequal variance (**A**-Ps.MAR).

To identify the cellular mechanisms underpinning the high–bone mass phenotype of *Tfeb*^CRa^ mice, we first performed dynamic histomorphometry of femurs from male *Tfeb*^CRa^ and Cre control mice. In this methodology, mice are injected with fluorochromes, which are incorporated into newly formed bone matrix. Mice are injected with 1 or more fluorochromes with specific intervals (e.g., 3 days and 7 days before sacrifice), which allows measurements of actively mineralizing surface (MS), mineral apposition rate (MAR), and bone formation rate (BFR) (40). *Tfeb* elevation in the osteoblast lineage increased bone formation at the periosteal surface (outer surface, Figure 5, A and B) but not at the endosteal surface (inner surface, Supplemental Figure 2D). This increase in periosteal bone formation was due to increased MS and MAR (Figure 5, A and B), suggesting that *Tfeb* elevation increases the number and rigor of periosteal osteoblasts. At the trabecular compartment, the increase in femoral trabecular bone volume of *Tfeb*^CRa^ mice was associated with increased MAR (Figure 5C and Supplemental Figure 2E). Together, these results suggest that *Tfeb* elevation in Osx1-Cre–targeted cells increases bone formation in both the cancellous and cortical compartments of femur.

To assess if bone formation was similarly increased in spine, we next measured expression of osteoblast marker genes in mRNA isolated from lumbar vertebrae. This analysis revealed higher levels of osteoblast marker genes (*Sp7*, Sp7 transcription factor 7/osterix; and *Col1a1,* collagen, type I, alpha 1) in *Tfeb*^CRa^ mice (Figure 5D), suggesting that *Tfeb* elevation also increases bone formation in spine. We also observed increased expression of osteoclast marker genes (*Acp5,* acid phosphatase 5, tartrate resistant; and *Ctsk*, cathepsin K) (Figure 5E). These results suggest a high-turnover state that favors bone formation in spine.

In line with these in vivo findings, elevation of *Tfeb* increased mineral deposition and proliferation in cultured osteoblastic cells (Figure 5, F and G). Sclerostin is a Wnt signaling inhibitor that is produced mainly by osteocytes to inhibit bone formation. Previous work has suggested that sclerostin is degraded in lysosomes (41). This raises the possibility that sclerostin levels could be altered in *Tfeb*^CRa^ mice. We examined this possible mechanism by quantifying sclerostin levels in serum and cortical bone, but we did not detect any differences between *Tfeb*^CRa^ and Cre control mice (Figure 5, H and I). Together, these results suggest that elevation of *Tfeb* in Osx1-Cre–targeted cells increases bone formation, likely by promoting the proliferation of osteoblast lineage cells.

### Elevation of Tfeb in osteoblast lineage cells is anabolic up to 12 months of age.

**Figure 6:**
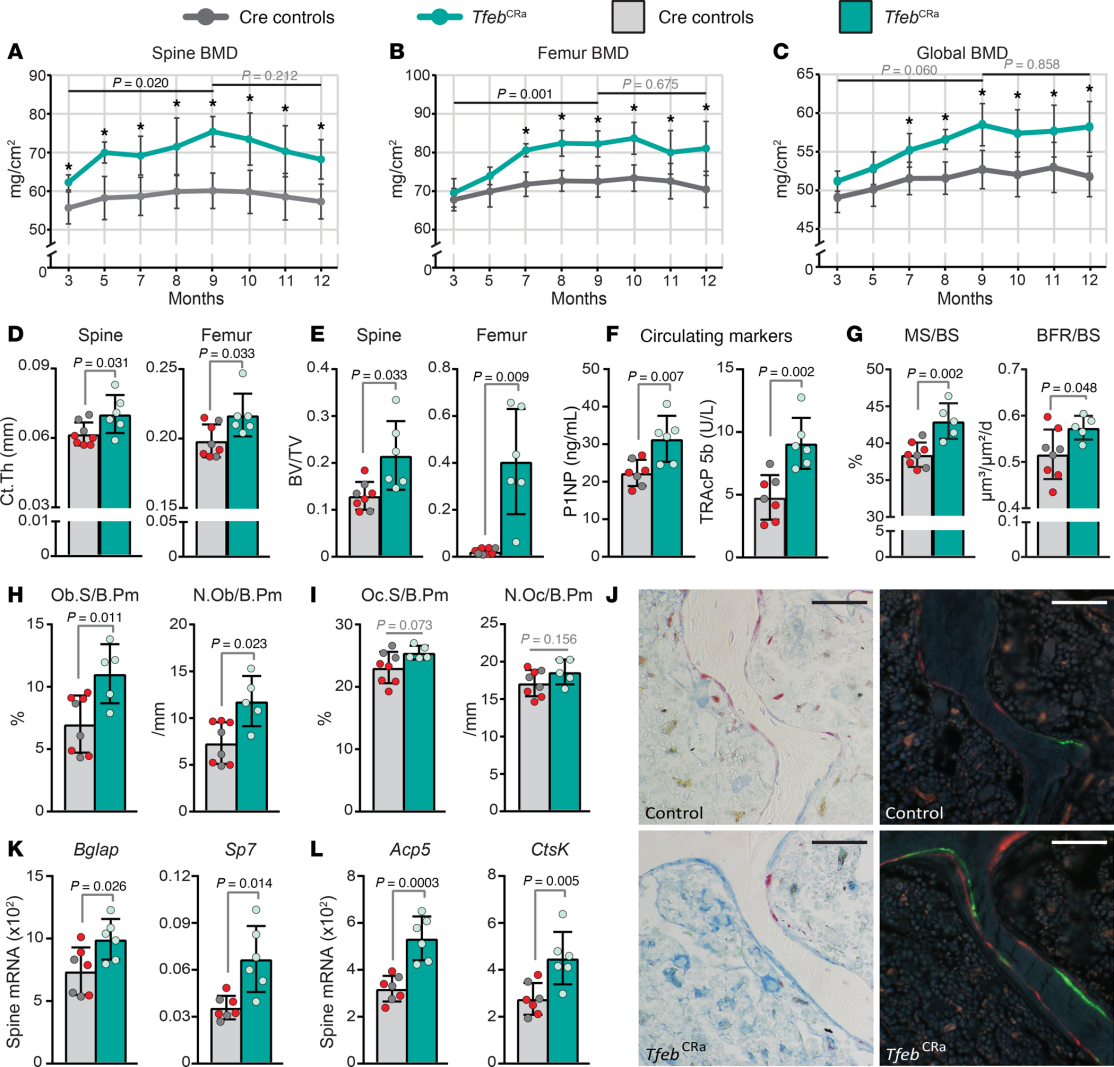
*Tfeb* elevation in the osteoblast lineage remains anabolic at 12 months of age. (**A**–**C**) Serial spine (**A**), femur (**B**), and global (whole body) (**C**) DXA BMD measurements were collected from female *Tfeb*^CRa^ mice (*n* = 6) and their littermate Cre controls (Osx1-Cre only, Osx1-Cre sgRNA*^Tfeb^* mice, *n* = 8) between 3 and 12 months of age. Error bars indicate SD. *P < 0.05 as calculated by unpaired *t* test at each time point. Indicated *P* values were calculated by repeated measures model (detailed in the Methods section) comparing the difference in genotypes at 3 versus 9 months or 9 versus 12 months. (**D** and **E**) μCT analysis of Ct.Th (**D**) and trabecular BV/TV (**E**) performed on lumbar vertebrae 4 (spine) and femurs from 12-month-old female *Tfeb*^CRa^ mice (*n* = 6) and littermate controls (Osx1-Cre only [red dots], Osx1-Cre sgRNA*^Tfeb^* [gray dots], *n* = 8). (**F**) ELISA measurements of P1NP and TRAcP 5b (*Tfeb*^CRa^ *n* = 6, controls *n* = 7). (**G**–**J**) Histomorphometric analysis of vertebral trabecular bone of *Tfeb*^CRa^ mice (*n* = 5) and their Cre controls (*n* = 8). (**G**) MS/BS, BFR/BS. (**H**) Osteoblast surface per bone perimeter (Ob.S/B.Pm), osteoblast number per bone perimeter (N.Ob/B.Pm). (**I**) Osteoclast surface per bone perimeter (Oc.S/B.Pm), osteoclast number per bone perimeter (N.Oc/B.Pm). (**J**) TRAcP 5b– and toluidine blue–stained or unstained (labeled with calcein and alizarin red) sections imaged with 40× original magnification. Scale bars represent 50 μm. (**K** and **L**) *Bglap* and *Sp7* (**K**) and *Acp5* and *Ctsk* (**L**) mRNA levels measured in lumbar vertebrae 5 (spine) of *Tfeb*^CRa^ mice (*n* = 7) and controls (*n* = 6) using qRT-PCR and normalized to mouse *Actb*. Bars indicate mean ± SD. Indicated *P* values were calculated by unpaired *t* test for equal or unequal variance (**E**-femur and spine, **K**-*Sp7*) or rank sum test (if data are not normally distributed, **D**-femur, **H**–N.Ob/B.Pm).

To assess the progression of the bone phenotype, we next performed serial analysis of bone mineral density (BMD) in live mice using dual-energy x-ray absorptiometry (DXA) from 3 to 12 months of age. *Tfeb*^CRa^ mice exhibited high spine and femur BMD beginning at 3 and 7 months, respectively, that remained high up to the time of sacrifice at 12 months (Figure 6, A and B). To address if elevation of *Tfeb* increased bone mass after rapid growth, we compared BMDs at 3, 9, and 12 months of age and showed that the high–bone mass phenotype became more pronounced up to 9 months of age and remained elevated up to 12 months of age (Figure 6, A–C).

MicroCT analysis performed at 12 months of age showed that *Tfeb*^CRa^ mice maintained elevated cortical thickness in both the spine and femur (Figure 6D). At this age (12 months), *Tfeb*^CRa^ mice had elevated trabecular bone volume compared with Cre controls (Figure 6E and Supplemental Figure 3, A and B). Of note, vertebral trabecular bone volume of female *Tfeb*^CRa^ mice was higher than Cre controls at 12 months of age (Figure 6E), but not at 5 months of age (Supplemental Figure 2B), suggesting that elevated *Tfeb* causes a progressive increase in vertebral trabecular bone volume. Mice lose femoral trabecular bone before age-related bone loss occurs in other sites or compartments (42–44). Specifically, by the time mice are 12 months of age, they have already lost most of their femoral trabecular bone (42–44). Consistent with this, Cre control mice had less than 4% trabecular bone volume at 12 months of age (Figure 6E). In contrast, at this age, *Tfeb*^CRa^ mice had approximately 40% trabecular bone volume, demonstrating that *Tfeb*^CRa^ mice retained their femoral trabecular bone volume up to 12 months of age (Figure 6E and Supplemental Figure 3B).

We next sought to examine the cellular basis of the high–bone mass phenotype of *Tfeb*^CRa^ mice at 12 months of age. Procollagen type 1 intact N-terminal propeptide (P1NP), a by-product of collagen processing by osteoblasts, can be detected in circulation and is used as a marker of bone formation (45). Tartrate-resistant acid phosphatase isoform 5b (TRAcP 5b), an enzyme synthesized and secreted by osteoclasts, can be detected in circulation and is used as a biochemical marker of osteoclast activity and bone resorption (45). Circulating P1NP and TRAcP 5b were both elevated in the serum of *Tfeb*^CRa^ mice, suggesting a high-turnover state (Figure 6F). However, as high bone volume may contribute to elevation of circulating markers, we performed further analysis of bone formation and resorption.

Dynamic histomorphometry in femurs revealed that at 12 months of age, periosteal and endosteal bone formation of *Tfeb*^CRa^ and Cre control mice were similar (Supplemental Figure 3, C and D). This suggests that while *Tfeb*^CRa^ mice maintain elevated cortical thickness and periosteal perimeter compared to controls, periosteal bone apposition does not remain elevated. As control mice do not have femoral trabecular bone at this age, a comparison of trabecular bone formation in femurs is not possible. However, as can be seen in representative images shown in Supplemental Figure 3E, bone formation is ongoing at the femoral trabecular compartment in *Tfeb*^CRa^ mice.

Next, we examined the cellular basis of high trabecular bone volume in spines of 12-month-old *Tfeb*^CRa^ mice. Histomorphometry revealed that *Tfeb* elevation increased MS and BFR (Figure 6, G and J) and increased osteoblast number and surface (Figure 6, H and J). Accordingly, expression of osteoblast marker genes (bone gamma-carboxyglutamate protein, *Bglap*; and *Sp7*) was increased in lumbar vertebrae of *Tfeb*^CRa^ mice (Figure 6K). *Tfeb* elevation in Osx1-Cre–targeted cells did not significantly change osteoclast number or surface (Figure 6I). Expression of osteoclast marker genes (*Acp5* and *Ctsk*) was also elevated in whole lumbar vertebrae (Figure 6L), suggesting that the higher bone volume of *Tfeb*^CRa^ mice is not due to decreased bone resorption. Together, these results suggest that *Tfeb* elevation in the osteoblast lineage increases osteoblast number and bone formation to cause a progressive increase in the vertebral trabecular bone volume of *Tfeb*^CRa^ mice.

### Tfeb elevation in Osx1-Cre–targeted cells promotes osteoblast production and function.

**Figure 7:**
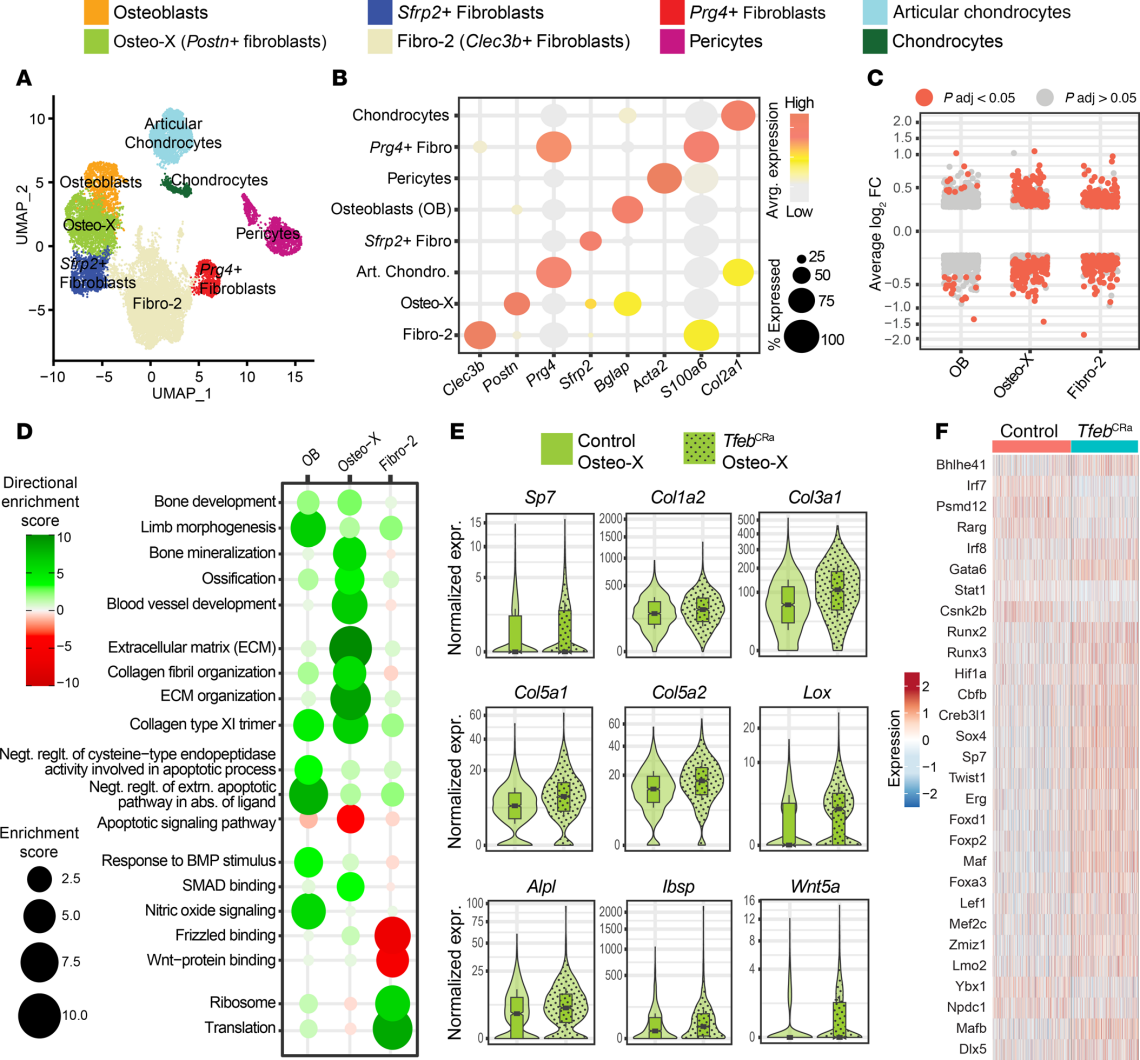
*Tfeb* elevation promotes production and function of osteoblasts. Periosteal mesenchymal cells isolated from femurs of 4-month-old female *Tfeb*^CRa^ mice and Cre littermate controls (Osx1-Cre CRa and Osx1-Cre sgRNA*^Tfeb^*) were subjected to scRNA-Seq using the 10X Genomics Chromium platform. *n* = 2 mice per group. (**A**) UMAP visualization of cell clusters obtained from the periosteum. (**B**) Dot plot of the representative marker genes for each cluster. The dot size indicates the percentage of cells expressing each gene, and the color gradient indicates expression level. (**C**) Volcano plots of genes significantly up- or downregulated in each cluster. (**D**) Biological processes significantly upregulated (green) or downregulated (red) in response to *Tfeb* stimulation as determined by GO analysis (*p*_adjusted < 0.05). Larger circle sizes and darker colors indicate higher significance. (**E**) Violin plots with box and whiskers representing the expression of select genes in Osteo-X cells. Wider parts of the violin indicate more data points. Inner box plots indicate the median (the line), quartiles (the boxes), and range of the data (whiskers). (**F**) SCENIC analysis of transcription factor activity in Osteo-X cells. UMAP, uniform manifold approximation and projection; BMP, bone morphogenetic protein; Lox, lysyl oxidase; Alpl, alkaline phosphatase; Ibsp, bone sialoprotein.

To identify mechanisms underpinning the high bone formation of *Tfeb*^CRa^ mice, we performed single-cell RNA-sequencing (scRNA-Seq) analysis of periosteal mesenchymal cells isolated from 4-month-old male *Tfeb*^CRa^ mice and their Cre controls. We chose to focus on the periosteum of *Tfeb*^CRa^ mice because this is the skeletal site that exhibited the most robust increase in bone formation in young mice (Figure 3D). Clustering analysis revealed 8 major clusters (Figure 7, A and B), namely osteoblasts, Osteo-X, *Sfrp2*^+^ fibroblasts, Fibro-2, *Prg4^+^* fibroblasts, pericytes, articular chondrocytes, and chondrocytes. Our genetic maneuver did not cause major changes in the relative abundance of these mesenchymal cell populations (Supplemental Figure 4A).

We have previously shown that osteoblasts, Fibro-2, and Osteo-X cells are targeted by the Osx1-Cre transgene (46). Therefore, we focused on the molecular impact of *Tfeb* elevation on these 3 mesenchymal cell populations. Model-based Analysis of Single-cell Transcriptomics (MAST) with a random effect for the sample of origin revealed that in the periosteum, *Tfeb* elevation in Osx1-Cre–targeted cells had the most impact in Fibro-2 and Osteo-X cells (Figure 7C). To examine the biological processes and cellular components that are altered by *Tfeb* elevation, we next performed gene set enrichment analysis (GSEA) in osteoblasts, Osteo-X, and Fibro-2 cells.

Gene ontology (GO) terms related to bone development, limb morphogenesis, ossification, and ECM synthesis organization were increased in *Tfeb*^CRa^ osteoblasts (Figure 7D). These changes were accompanied by the elevation of genes related to ribosome and translation (Figure 7D). Together, these results suggest an increase in osteoblast function. *Tfeb*^CRa^ osteoblasts also exhibited elevated BMP and nitric oxide signaling and reduced apoptotic signaling pathways (Figure 7D). These pathways are influenced by autophagy in other tissues (47–49). Further studies are needed to address how *Tfeb* elevation and autophagy impact these pathways in osteoblasts and their functional contribution to the high bone formation of *Tfeb*^CRa^ mice.

Cells of the Osteo-X cluster highly express periostin (*Postn*) (46). Expression of *Postn* and *Postn*-expressing cells increase in response to fracture (50). However, the function or fate of Osteo-X cells under physiological or pathological conditions is unclear. Herein, we show that Osteo-X cells of *Tfeb*^CRa^ mice exhibited significant increases in GO terms related to bone development, bone mineralization, ossification, collagen-containing ECM synthesis, and ECM organization (Figure 7D). Specifically, Osteo-X cells of *Tfeb*^CRa^ mice exhibited elevated expression of several markers of osteoblast differentiation (Figure 7E and Supplemental Figure 4B) such as osteoblastic transcription factors (e.g., *Sp7*), various constituents of bone matrix including various collagens (e.g., *Col1a2*, *Col3a1*, *Col5a1*, and *Col5a2*), enzymes necessary for collagen organization (e.g., *Lox*), noncollagen components of bone matrix (e.g., *Ibsp*), and genes related to bone matrix mineralization (e.g., *Alpl*). These changes in Osteo-X cells were associated with increased BMP and Wnt signaling pathways (Figure 7D). Therefore, we hypothesize that Osteo-X cells are periosteal osteoblast progenitors and that in *Tfeb*^CRa^ mice, these cells commit to becoming osteoblasts, perhaps in response to elevated Wnt and BMP signaling pathways. In contrast, Fibro-2 cells exhibited only modest changes that would be indicative of their commitment to becoming osteoblasts (Figure 7D).

*Wnt5a* expression in Osx1-Cre cells supports osteoblast number and bone formation, likely by enhancing Wnt and BMP signaling (51, 52). Therefore, increased *Wnt5a* in Osteo-X cells of *Tfeb*^CRa^ mice (Figure 7E) may be potentiating Wnt and BMP pathways to stimulate bone formation.

Single-Cell Regulatory Network Inference and Clustering (SCENIC) analysis of transcription factor activity in Osteo-X cells (Figure 7F) revealed increase in activity of RUNX family transcription factor 2 (RUNX2) and RUNX3, accompanied by decreased STAT1 activity (a RUNX2 inhibitor) (53) and elevated activity of RUNX2 activators CBFB (54) and DLX5 (55). Based on known interactions and their roles in osteogenesis, high RUNX2 and RUNX3 activity along with increased activities of SP7 (56), DLX5 (55), SOX4 (57, 58), HIF-1α (59), CREB3L1 (60), MAF (61), and LEF1 (62) likely stimulate osteogenesis of *Tfeb*^CRa^ mice (Figure 7F and Supplemental Figure 5). Further studies are required to establish a functional connection among these transcription factors, autophagy, and bone formation of *Tfeb*^CRa^ mice.

## Discussion

We and others have previously shown that autophagy in osteoblast lineage cells is essential for bone formation (4–8). However, whether stimulation of autophagy would have a beneficial impact on bone was unknown. In the present study, we aimed to address this question by stimulating autophagy via elevating the expression of endogenous *Tfeb*, a master transcriptional regulator of autophagy and lysosomal biogenesis, in the entire osteoblast lineage. Using multiple methodologies, we verified that elevation of *Tfeb* in osteoblast lineage cells increases the autophagic flux of osteoblasts. Moreover, we showed that elevation of *Tfeb* in the osteoblast lineage results in a skeletal and cellular phenotype that is the opposite of autophagy elimination in this lineage (5). These results suggest that the anabolic impact of *Tfeb* elevation in the osteoblast lineage is due to stimulation of autophagy.

We used CRISPRa for our gain-of-function studies. This methodology has various benefits over traditional transgene-based overexpression approaches. In traditional approaches, transgene expression is influenced by copy number, integrity, and insertion site into the genome (63–66). Transgenic approaches can also lead to undesirably high expression levels, causing unwanted changes in transcription (24). In contrast, CRISPRa stimulates the transcription of endogenous genes, which avoids the copy number and integration site effects. CRISPRa also provides control over the magnitude of gene stimulation. Herein, we show that expression of an sgRNA from a safe harbor locus is sufficient to elevate target gene expression in vivo via CRISPRa. In traditional transgenic approaches, transgene expression may also be suppressed over time (63–65). In contrast, herein we show that transcriptional stimulation via CRISPRa persists up to at least 12 months of age (Figure 1D). Overall, our results demonstrate that CRISPRa can effectively and reliably stimulate target genes in a cell type–specific manner in mice.

Our genetic maneuver, elevation of endogenous *Tfeb*, increased trabecular bone volume in femur earlier than spine. Specifically, while the increase in trabecular bone volume and trabecular number was observed in long bones at 5 months of age (Figure 3), it became evident in spine only at 12 months of age (Figure 6 versus Figure 3). This may be due to differences in turnover of trabecular bone compartment in long bones versus spine. Specifically, Glatt et al. (44) previously showed that trabecular volume is lost at a higher rate in long bones compared with spine, especially between 2 and 6 months of age. This suggests that in long bones, bone remodeling favors resorption and causes loss of bone in every remodeling cycle, even in young mice. Therefore, it is likely that increasing bone formation in such a setting shifts the balance of remodeling and thereby causes a more dramatic phenotype in long bones.

Our scRNA-Seq analysis suggests that *Tfeb* elevation increases osteoblast function and survival. Collagen is thought to have a propensity to misfold (67). Accordingly, as osteoblasts differentiate and start producing collagen, the unfolded protein response increases (68–70). Autophagy clears misfolded collagen in osteoblasts (71), and lack of autophagy was shown to induce ER stress (8). Therefore, *Tfeb* elevation may stimulate autophagy to improve protein homeostasis and enhance the capacity of osteoblasts to form bone. In addition, we observe a decrease in osteoblast apoptosis. Previous studies established that reduction of osteoblast apoptosis is sufficient to increase bone formation and bone mass (72). Therefore, further studies are needed to assess the contribution of apoptosis to the increase in bone formation of *Tfeb*^CRa^ mice.

Based on our unbiased scRNA-Seq analysis (Figure 7), we propose that Osteo-X cells are periosteal osteoblast progenitors and that elevation of *Tfeb* in Osteo-X cells promotes their osteoblastic differentiation. Nonetheless, future lineage tracing and functional studies are needed to test this hypothesis and assess their functional contribution to the elevated bone formation of *Tfeb*^CRa^ mice.

Our ex vivo studies suggest that elevated proliferation can be increasing osteoblast numbers in *Tfeb*^CRa^ mice. However, we did not detect significant changes in proliferation-related genes (e.g., *Top2a*, *Mki67*) or GO terms in our scRNA-Seq analysis. One explanation for this may be the difference in osteoblast progenitors in periosteum versus bone marrow environment. In recent years, different groups have identified different osteoblast progenitor populations residing in the bone marrow using combinations of scRNA-Seq and lineage tracing (73–75). However, there is not yet a consensus about the exact identity or function of these different cell populations. Future studies using scRNA-Seq of the endosteal cells and in vivo 5-ethynyl-2′-deoxyuridine (EdU) labeling are needed to establish changes in specific cell populations and the level of progenitor proliferation in *Tfeb*^CRa^ mice.

In skeletal muscle, TFEB regulates mitochondrial biogenesis and function by increasing expression of genes involved in these processes (76). In contrast, osteoblasts, Osteo-X and Fibro-2 cells of *Tfeb*^CRa^ mice have decreased expression of genes associated with mitochondrion, ATP biosynthetic process, and mitochondrial membrane potentials (Supplemental Figure 6). This suggests that an increase in mitochondrial biogenesis is not a likely contributor to the increase in bone formation of *Tfeb*^CRa^ mice.

Dysfunctional autophagy is a hallmark of aging (77). Several studies examined the impact of autophagy stimulation in the setting of aging or age-related pathologies and showed that genetic stimulation of autophagy increases proliferation and restrains other hallmarks of aging, such as mitochondrial dysfunction, loss of proteostasis, and cellular senescence (14, 78–81). In light of evidence that autophagy declines with age in osteoblast lineage cells (9–11), and that loss of autophagy mimics several aspects of skeletal aging (4), we propose that declining autophagy could be contributing to age-related bone loss. If this is the case, stimulation of autophagy may blunt age-related bone loss. To test this, others have administered rapamycin to old mice (16- and 20-month-old) (9, 82) and rats (24-month-old) (83) with varying doses and intervals. While 2 of these studies showed a beneficial impact on trabecular bone, the other did not demonstrate any effect on trabecular or cortical bone. Part of the discrepancy between these studies may be due to differences in dose and interval of administration or age or sex of the model organism. However, as stated above, all 3 studies are confounded by the lack of cellular and molecular specificity of this mTOR inhibitor. Thus, *Tfeb*^CRa^ mice will be a valuable tool for determining whether declining autophagy in cells of the osteoblast lineage is sufficient to prevent any aspects of skeletal aging.

In conclusion, increasing endogenous *Tfeb* expression in the osteoblast lineage promotes autophagy, and bone formation, and leads to a remarkable increase in bone mass. These findings lay the groundwork for future studies aimed at examining whether stimulation of autophagy can blunt skeletal pathologies associated with insufficient autophagy or decreased bone formation.

## Methods

### Sex as a biological variable.

We considered sex as a biological variable, conducted our studies in both female and male mice, and analyzed the effect of our maneuver in both male and female mice. We did not observe sex-related differences in bone mass or architecture in response to our genetic maneuver. Therefore, the findings are expected to be relevant for both sexes.

### Testing Tfeb sgRNAs in vitro.

Five candidate sgRNAs were identified using the Zhang Laboratory CRISPR Design tool (http://crispr.mit.edu) by searching the region 150 bp upstream and downstream from the *Tfeb* TSS. sgRNAs with high-quality scores, minimal off-target scores, and no potential off-targets in other genes were chosen as candidates. pX330 (84) (Addgene plasmid 42230) and pX458 (85) (Addgene plasmid 48138) were obtained from the Zhang Laboratory via Addgene. The Cas9 sequence of the pX330 plasmid was replaced with enhanced GFP subcloned from the px458 plasmid producing the sgRNA_GFP plasmid. Each of the 5 candidate *Tfeb* target sgRNA sequences indicated below was cloned into an sgRNA_GFP plasmid producing *Tfeb* sgRNA-1 through -5. Sequences for the sgRNAs are as follows: sgRNA-1: AAATCCCGGCGAGCCCTTCG (protospacer adjacent motif [PAM]: CGG), sgRNA-2: ACATTTCCCAGCGGGCACAG (PAM: CGG), sgRNA-3: CGGGACGCAGAGAACGGAGA (PAM: CGG), sgRNA-4: AACGGAGACGGCGCCGACAG (PAM: CGG), and sgRNA-5: TGCCGGGCGCAGCGGGAAGT (PAM: GGG). Each *Tfeb* sgRNA plasmid was cotransfected with SP-dCas9-VPR plasmid (21) (a gift from George Church, Department of Genetics, Harvard Medical School, Boston, Massachusetts; Addgene plasmid 63798) into UAMS-32 cells (86) (a gift from Charles A. O’Brien, University of Arkansas for Medical Sciences) using PolyJet reagent (SignaGen Laboratories). After 48 hours, transfected cells were subjected to fluorescence-activated cell sorting to acquire GFP-positive cells, which contain CRISPRa components, and sorted into 3 wells of a 96-well plate at a density of 7,500 cells per well. Cells were incubated overnight, and then, Cells-to-C_T_ kit (Invitrogen, A25605) was used to harvest RNA and measure gene expression via qRT-PCR.

### Generation of mice.

The sgRNA*^Tfeb^* mice were produced via CRISPR/Cas9-mediated knockin of a U6-sgRNA*^Tfeb^*_4 cassette into the Rosa26 locus as previously described (25, 87). Founders were screened for the presence of the knockin sequence using F1 5′-AAGCACTTGCTCTCCCAAAG-3′; R1 5′-GGCGGATCACAAGCAATAAT-3′. The knockin was confirmed by DNA sequencing. The progeny were genotyped using genomic DNA isolated from the tail tips using F1, R1, F2 5′-GAGGGCCTATTTCCCATGAT-3′; R2 5′-GGTGTTTCGTCCTTTCCACA-3′ primers. CRa (22) and Osx1-Cre (26) mice were previously produced by Zhou et al. and Rodda et al., respectively. These mice were obtained from and genotyped according to the directions of The Jackson Laboratory (Strain 0331645 and Strain 006361). All mice were provided food and water ad libitum and were maintained on a 12-hour light/12-hour dark cycle.

### Animal experiments.

Mice carrying the Osx1-Cre transgene are known to be smaller and have low bone mass when compared with mice without this transgene (88). These effects are minimized or eliminated when expression of the transgene is suppressed by doxycycline (89). Therefore, we utilized doxycycline to suppress expression during development and until 3 weeks of age (26, 88). Specifically, we maintained breeders and their progeny (up to 3 weeks of age) on a doxycycline-containing diet (Figures 3–7), which prevents Cre expression and bypasses the impact of the Osx1-Cre transgene on skeletal development (89). Nonetheless, to account for any effects of the Osx1-Cre transgene as mice age, we compared *Tfeb*^CRa^ mice with their Cre-harboring littermates. Mice were then aged to 5 to 12 months of age, and detailed skeletal analysis was performed (Figures 3–7).

### Ex vivo osteoblastic cell culture.

For ex vivo mineral deposition assay and protein isolation, bone marrow cells were flushed from tibias and femurs of mice and cultured in osteogenic media as previously described (5). The cells were seeded into 6- or 12-well plates at 12.5 × 10^6^ cells/well or 5 × 10^6^ cells/well densities, respectively. After 21 days, protein was isolated for downstream analysis, and ex vivo mineral deposition assay was performed. Briefly, the cultures were fixed with 50% ethanol at 4°C for 15 minutes, dried for 2 hours, and stained with an aqueous solution of 40 mM alizarin red for 30 minutes at room temperature.

For flow cytometry, confocal microscopy, and ex vivo proliferation assay, bone marrow cells were flushed from the tibias and femurs of mice using α-MEM culture media. Red blood cells were removed using ACK buffer (0.01 mM EDTA, 0.011 M KHCO_3_, and 0.155 M NH_4_Cl, pH 7.3). The remaining cells were cultured with osteogenic media. After cells reached 80% confluence (5 to 10 days), cells were replated in 6-well plates at 2.5 × 10^4^ cells/cm^2^.

### Ex vivo proliferation assay.

Cells were isolated from tibias and femurs of mice as indicated above and plated in 96-well cell culture plates at 5 × 10^4^ cells/cm^2^, in complete media. After 24 hours, media were replaced with media containing 100 μM BrdU. The plates were then incubated at 37°C and 5% CO_2_ for 24 or 48 hours. The cell proliferation assay was performed according to the manufacturer’s instructions (Roche Applied Sciences, 11647229001).

### Flow cytometry.

Cells isolated from murine long bones were incubated with 50 nM of LysoTracker (Invitrogen, L12492) or 200 nM of MitoTracker (Invitrogen, M7512) for 30 minutes, in α-MEM at 37°C and 5% CO_2_. Cells were then washed with PBS, resuspended in PBS with 0.5% BSA, and immediately analyzed by flow cytometry. Autophagy was measured using the autophagy detecting kit (Abcam, ab270790) with minor modifications to instructions. Briefly, cells were incubated in complete media or low-serum (2% FBS) media for 2 hours prior to staining. Then, cells were incubated in 0.5× Autophagy Probe prepared in complete or low-serum media for 30 minutes at 37°C and 5% CO_2_, washed with PBS, and fixed with provided kit fixative at a 1:10 dilution for 15 minutes. All samples were acquired using LSRFortessa (BD Biosciences) with FACSDiva software version 6.1.3. Data were analyzed using FlowJo 10.10 (Tree Star Inc.). Populations were first plotted according to their size and complexity on a forward scatter-A (FSC-A) versus side scatter-A (SSC-A) plot, to remove any dying cells and debris, followed by FSC-A versus FSC-H plot to isolate single cells. The excitation/emission settings were LysoTracker (Ex. 647 nm, Em. 668 nm), MitoTracker (Ex. 579 nm, Em. 599 nm), and Autophagy Probe (Ex. 590 nm, 620 nm).

### Confocal microscopy.

To image mitochondria, cells were plated at 2.5 × 10^4^ cells/cm^2^ in a 96-well plate with ibiTreat: #1.5 polymer coverslip surface modification (Ibidi, 89626). After 24 hours, cells were incubated with 200 nM of MitoTracker (Invitrogen, M7512) for 30 minutes, in fresh α-MEM with no FBS at 37°C and 5% CO_2_ in dark conditions. After treatment, cells were washed, and media were replaced with fresh medium containing Hoechst 33342 stain (Thermo Fisher Scientific, 62249) diluted 1:1,000 and imaged immediately. To image autophagosomes and autolysosomes, cells were plated on 8-well Lab-Tek Glass Chamber Slides at 3.75 × 10^4^ cells/cm^2^ (Thermo Fisher Scientific, 177402). Prior to staining, cells were incubated in complete media or low-serum (2% FBS) media for 2 hours. Then the cells were incubated for 30 minutes with 0.5× Autophagy Probe, Red (Abcam, ab270790), and Hoechst was prepared in complete or low-serum media. Cells were washed, and media were replaced with fresh PBS. MitoTracker- and Autophagy Probe–labeled cells were imaged using an LSM 880 confocal microscope (Carl Zeiss, Inc.) equipped with a Plan-Apochromat 63×/1.4 Oil DIC M27 objective or EC Plan-Neofluar 20×/0.30 M27 objective, respectively. Images were acquired using Zeiss ZEN 2.3 SP3 software. Hoechst was imaged using a 405 nm laser, and MitoTracker and Autophagy Probe were imaged using a 561 nm laser.

### RNA isolation and gene expression analysis.

Lumbar vertebrae 5 and calvaria were dissected, cleaned of soft tissue, snap-frozen in liquid nitrogen, and stored at –80°C. For RNA isolation, the bones were homogenized in TRIzol Reagent (Life Technologies, 15596018). RNA was isolated from homogenized bones with the RNeasy Plus Mini Kit (QIAGEN, 74136). A total of 1 μg RNA was used to synthesize cDNA with a High-Capacity cDNA Reverse Transcription Kit (Applied Biosystems, 4368814). Relative abundance of mRNAs was measured using multiplex qRT-PCR with TaqMan Fast Advanced Master Mix (Applied Biosystems, 4444964), FAM-labeled TaqMan gene expression assays (Thermo Fisher Scientific), and VIC-labeled mouse Actb (β-actin) (Applied Biosystems, 4352341E). The following FAM-labeled assays were used in the gene expression analysis: *Tfeb* (Mm00448968_m1), *Lamp1* (Mm01217070_m1), *CtsB* (Mm01310506_m1), *CtsD* (Mm00515586_m1), *CtsF* (Mm00490782_m1), *Clcn7* (Mm00442400_m1), *Gns* (Mm00659592_m1), *Hexa* (Mm00599877_m1), *Mcoln1* (Mm00522550_m1), *Tpp1* (Mm00487016_m1), *Map1lc3* (LC3, Mm00782868_sH), *Atg7* (Mm00512209_m1), *Becn1* (Mm01265461_m1), *Atg9b* (Mm01157883_g1), *Wipi1* (Mm00461219_m1), *Uvrag* (Mm00724370_m1), *Sqstm1* (Mm00448091_m1), *Parkin* (Mm01323528_m1), *Pink* (Mm00550827_m1), *Bnip3* (Mm01275600_g1), *Bnip3l* (Mm00786306_s1), *Mfn2* (Mm00500120_m1), *Fundc1* (Mm00511132_m1), *Sp7* (Osx, Mm04209856_m1), *Col1a1* (Mm00801666_g1), *Acp5* (Mm00475698_m1), *Ctsk* (Mm00484039_m1), and *Bglap* (OCN, custom, assay ID APCE4V6, primers F 5′-GCTGCGCTCTGTCTCTCTGA-3′, R 5′-TGCTTGGACATGAAGGCTTTG-3′, probe 5′-FAMAAGCCCAGCGGCC-NFQ-3′). The relative mRNA levels were calculated using the comparative cycle threshold method (90). One *Tfeb*^CRa^ sample in the 4.5-month-old male experiment, and 1 Cre control sample in the 12-month-old female experiment, had low 260/280 ratios, and the mRNA levels from these 2 samples were outliers (2 SDs away from the mean). Therefore these 2 samples were excluded from gene expression analysis presented in Figure 5, D and E, and Figure 6, K and L.

### Immunoblot analysis.

Protein was extracted from cultured cells or pulverized bone using RIPA Buffer (Thermo Fisher Scientific, PI89901) with protease/phosphatase inhibitors (Cell Signaling Technology [CST], 5872S). Proteins were resolved in 4%–20% or 4%–15% Mini-PROTEAN TGX gels (BIORAD, 4561093 and 4561083, respectively) and transferred onto TransblotTurbo midi-size nitrocellulose membranes (0.2 μm pore size, BIORAD, 1704271). The membranes were blocked for 5 minutes with EveryBlot Blocking Buffer (BIORAD, 12010020) and incubated overnight with primary antibodies, with rocking at 4°C. The primary antibodies used were TFEB (Invitrogen, PA5-96632, 1:500 dilution), LAMP1 (CST, 99437, 1:1,000 dilution), p62 (CST, 23214s, 1:1,000 dilution), LC3 (CST, 12741T, 1:1,000 dilution), Sclerostin (R&D Systems, AF1589, 1:500 dilution), and β-actin (MilliporeSigma, A5316, 1:4,000 dilution). After overnight incubation, membranes were washed 3 times with PBS for 5 minutes each and incubated for 45 minutes with appropriate secondary antibodies conjugated with IRDye 680 or IRDye 800 dyes (LI-COR, 1:2,000 dilution). After washing with PBS 3 times for 5 minutes each time, membranes were scanned and analyzed with an Odyssey IR imaging system (LI-COR) and Image Studio Software (Version 5.2).

### Skeletal phenotyping.

BMD was measured in live mice by DXA with a PIXImus Mouse Densitometer (GE Lunar Corp.) as previously described (91). Calvaria and shoulder blades were excluded from total-body BMD analysis. Vertebral BMD was measured in lumbar vertebrae 2–6, and femoral BMD was measured using the right femur.

For microCT analysis, lumbar vertebrae 5, femurs, and humeri were dissected; cleaned of soft tissue; wrapped in saline-soaked gauze; and stored at –20°C. Unlike the male experiment, which used femurs as long bones, in the female experiment humeri were used for microCT. This is because femurs were dissected and cut for isolation of bone marrow cells for ex vivo analysis. Samples were thawed before scanning and medium-resolution scans were obtained (12 μm isotropic voxel size) using a model μCT40 (Scanco Biomedical) as previously described (25). Long bone midshaft cortical measurements were performed by drawing contours to measure the cortical thickness on the 20 slices flanking the midshaft. Long bone trabecular analysis was performed by drawing contours every 20 slices of the distal metaphysis beginning 10 slices proximal to the growth plate and ending 151 slices proximal to the first contour. Vertebral cortical thickness and trabecular analyses were performed by drawing contours every 10 slices between the 2 growth plates of the vertebrae. The vertebral cortical bone thickness was determined on the ventral cortical wall using contours of cross-sectional images, drawn to exclude trabecular bone.

### Biomechanical testing.

The mechanical properties of the femur were determined by a 3-point bending analysis using ElectroForce 5500 (TA Instruments) as previously described (92). Femurs were placed posterior-side down on a miniature bending apparatus with lower supports set at 8 mm apart with the left support set proximal to the distal condyles. Load was applied to the anterior surface of the femur midway between the lower supports. The load was applied at a constant rate of 3 mm/min until failure. Structural or extrinsic properties and material or intrinsic properties were derived by measuring the maximum load and displacement of the femur and normalized for bone size. The external measurements of the femora were recorded with a digital caliper. Bone geometry and volume were determined by microCT. During 3-point-bending analysis of 4.5-month-old male mice, the femur of 1 *Tfeb*^CRa^ mouse slipped, rendering the analysis invalid, thereby prompting us to not include this mouse in our analysis (Figure 4).

### Histology.

Mice were injected with 20 mg/kg of calcein (Sigma-Aldrich, C0875) and alizarin complexone (Sigma-Aldrich, A3882), 6 days and 2 days before euthanasia, respectively. For histology, femurs or L1–3 vertebrae were fixed in 10% Millonig’s formalin for 24 hours, then dehydrated using a series of serial dilutions of ethanol into 100% ethanol. Next, the bones were embedded in methyl methacrylate, and longitudinal sections were obtained. Histomorphometric measurements were performed as previously described (40, 93). During dynamic histomorphometric analysis of 12-month-old female mice, we noticed that 1 *Tfeb*^CRa^ mouse was missing 1 of the 2 fluorochrome injections, since no double labels were observed in femur or spine. This mouse was excluded from the analysis shown in Figure 6, G–J, and Supplemental Figure 3, C and D.

### Serum bone turnover assays.

Blood was collected from the facial vein and incubated at room temperature for a minimum of 30 minutes to allow the blood to clot. Serum was collected by serial centrifugation (twice at 2,000*g* for 12 minutes) and stored at –20°C. P1NP and TRAcP 5b were measured following the manufacturer’s instructions (Immunodiagnostic Systems, AC-33F1 and SB-TR103). Circulating sclerostin levels were measured using Mouse/Rat SOST/Sclerostin Quantikine ELISA Kit (R&D Systems, MSST00). We had difficulty collecting blood from one 12-month-old Cre control mouse; serum from this mouse was little in amount and showed marked hemolysis. Therefore, this sample was not included as part of the analysis shown in Figure 6F.

### Cell isolation for 10X Genomics sequencing.

Periosteal cell isolation was performed as previously described (46). Briefly, femurs from 2 mice per group were cleaned up of muscle without damaging the periosteum. Intact femurs were exposed to 3 collagenase digestions and 3 EDTA decalcifications. Isolations were enriched in mesenchymal cells by depletion of hematopoietic and endothelial cells (46). Enriched cells were encapsulated using a Chromium Controller (10X Genomics); libraries were constructed using a Chromium Single Cell 3’ Reagent Kit (10X Genomics) and sequenced using Illumina NovaSeq 6000 to generate FASTQ files.

### Bioinformatic analysis of scRNA-Seq.

The FASTQ files were preprocessed using Cell Ranger software version 7.1 (10X Genomics) to produce feature-barcode matrixes. Mouse reference genome mm10 was used for alignments. The feature-barcode matrixes were imported for further analysis in R suite software using Seurat package version 4.2.0 (94). Cells containing between 1,000 and 5,000 transcripts, mitochondria read content less than 15%, and complexity of RNA species (log_10_[number of genes/number of unique molecular identifiers] > 0.85) were included in the further analysis. Harmonization was performed using a reciprocal principal component analysis method based on the top 50 principal components and 6,000 most variable features to minimize batch effect. Harmonized results were used for clustering using Louvain algorithm with multilevel refinement and UMAP for dimension reduction. The gene-specific markers of individual clusters and differentially expressed genes were identified using MAST algorithm for cell type identification (95). The GSEAs were performed using piano package (96). Transcriptional activity in osteoblasts (Supplemental Figure 4C) and in Osteo-X cells (Figure 7) was analyzed using SCENIC pipeline software (97). Interaction among the identified transcription factors was examined using STRING database v12.0 (98). The network was then imported to Cytoscape software (https://cytoscape.org/) for visualization.

### Statistics.

For 2-sample analyses evaluating differences between Cre controls and *Tfeb*^CRa^, data distributions were checked for normality and equal variance to choose the appropriate test for each 2-tailed *t* test (equal variances), Welch’s *t* test (unequal variances), or Wilcoxon’s rank-sum test (non-normal data). ANOVA was employed for analyses of more than 2 groups, checking for normality and homoscedasticity of the residuals and transforming the data if needed to meet those assumptions. One-way ANOVA was used for Figure 1B and 2-way ANOVA for Figure 2, B–E. A *P* < 0.05 was considered significant. For Figure 6, a repeated measures model was fit to BMD measurements focusing on the effects of group and month, along with the interaction of group and month and with an autoregressive (1) covariance structure using SAS version 9.4. Residuals of the model were tested for normality and constant variance.

### Study approval.

All animal studies were carried out with approval from and following the policies of the Institutional Animal Care and Use Committee of the UAMS. The studies described here were performed and reported in accordance with Animal Research: Reporting of In Vivo Experiments guidelines. Each figure legend indicates the sex, number, and age of the mice used for each experiment.

### Data availability.

The raw data are contained in the Supporting Data Values file and in NCBI BioProject RJNA1238393 (scRNA-Seq).

## Author contributions

MO conceived and designed the in vivo experiments. MA and MO designed the in vitro experiments. MO, AJ, JAH, IK, JL, and JC performed all animal experimentation and skeletal analysis. MO, AJ, and AMC generated and analyzed the in vitro data. AYS performed 3-point bending analysis. JT, MO, and AJ performed statistical analysis. MO and JX performed periosteal cell isolation, IN and VW performed scRNA-Seq and advanced bioinformatics analysis. MO and AJ prepared figures. MO wrote the manuscript. All authors reviewed the manuscript.

## Supplementary Material

Supplemental data

Unedited blot and gel images

Supporting data values

## Version 1 07/29/2025

In-Press Preview

## Version 2 09/09/2025

Electronic publication
