# Supplementary material for: Elevation of master autophagy regulator Tfeb in osteoblast lineage cells increases bone mass and strength
Source: JCI Insight. 2025 Jul 29;10(17):e191688. doi: 10.1172/jci.insight.191688 (PMC12487674; doi:10.1172/jci.insight.191688)
Supplement: Supplemental data [file jciinsight-10-191688-s248.pdf]

Supplementary Materials

Elevation of master autophagy regulator *Tfeb* in osteoblast lineage cells increases bone mass and strength

Alicen James, James Hendrixson, Ilham Kadhim, Adriana Marques-Carvalho, Jacob Laster, Julie Crawford, Jeff Thostenson, Visanu Wanchai, Amy Y. Sato, Intawat Nookaew, Jinhu Xiong, Maria Almeida, and Melda Onal

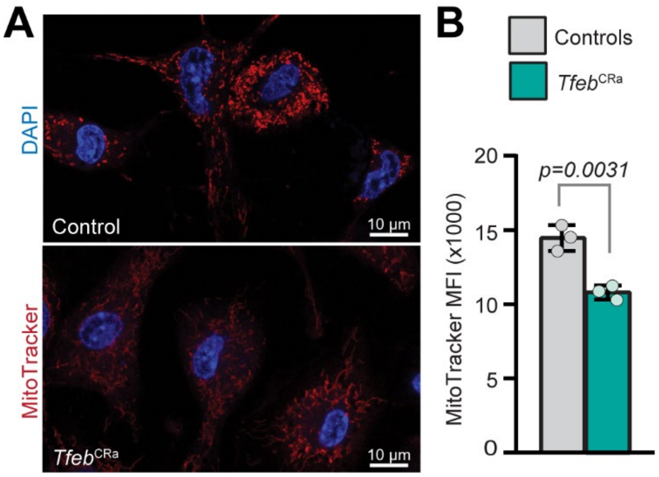

Supplemental Figure 1. *Tfeb* elevation in osteoblast lineage cells decreases mitochondrial content. Bone marrow cells were isolated from femurs and tibias of 5-month-old female *Tfeb*^{CRa} mice and littermate controls and differentiated into osteoblasts using osteogenic media. Cells were then stained with MitoTracker. (**A**) Representative confocal micrograph, scale bars represent 10 µm. (**B**) Quantification of MitoTracker mean fluorescence intensity (MFI) measured via flow cytometry. n = 3 wells per group. Bars indicate mean $\pm$ SD. Indicated *p*-values were calculated by unpaired *t*-test.

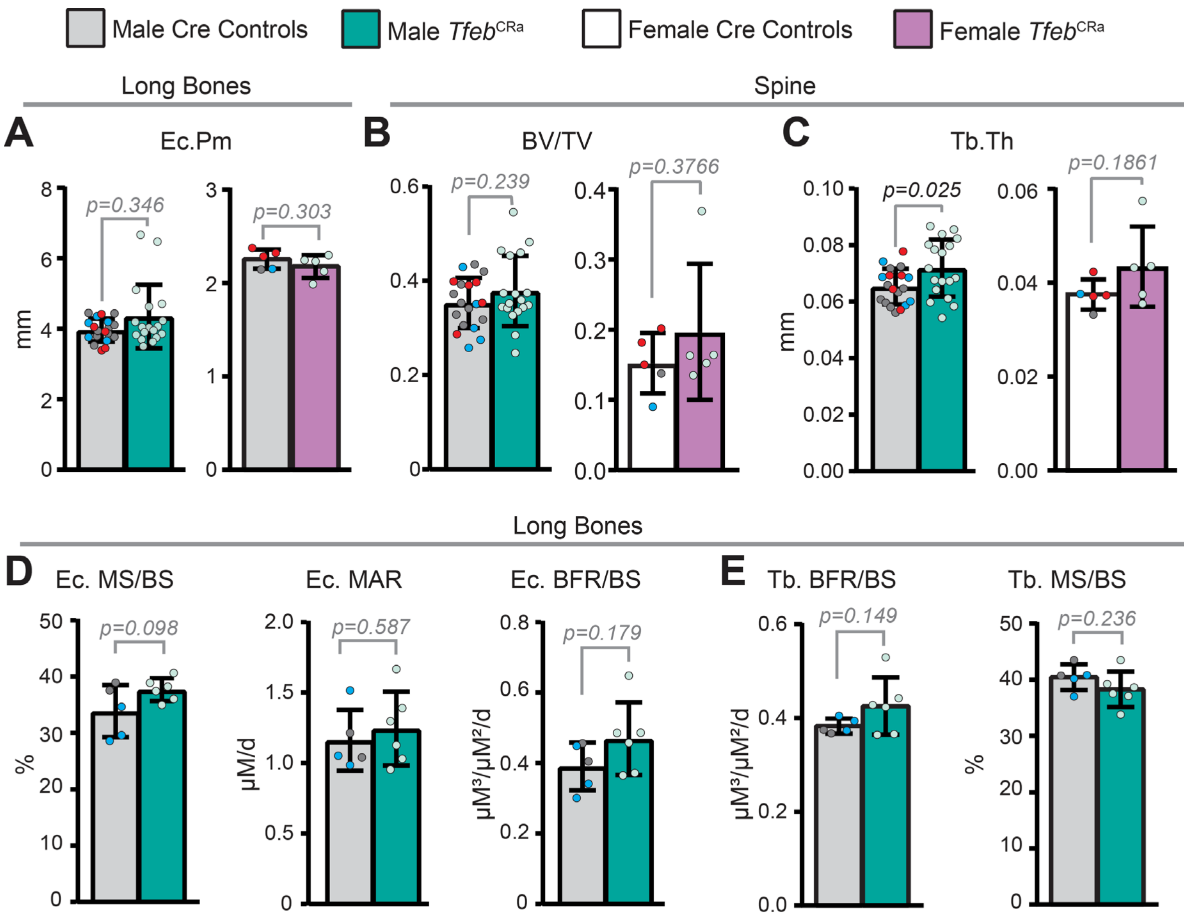

Supplemental Figure 2. *Tfeb* elevation does not impact femur endosteal bone or vertebral trabecular bone at 4.5 months of age. (**A-C**) µCT analysis was performed on long bones and spine (L4) of 4.5-month-old *Tfeb*^{CRa} mice and their Cre littermate controls (Osx1-Cre only [red dots)], Osx1-Cre;CRa [blue dots], Osx1-Cre;sgRNA^{Tfeb} [gray dots]). Male (M), n = 18-19 mice per group; Female (F), n = 5 mice per group. (**A**) Endosteal perimeter (Ec.Pm) was measured at the femur midshaft. (**B**) Trabecular bone volume over tissue volume (BV/TV) was measured in L4. (**C**) Trabecular thickness (Tb.Th) was measured in L4. (**D-E**) Dynamic histomorphometry was performed on femurs of *Tfeb*^{CRa} mice and their littermate controls. n = 5-6 mice per group. (**D**) Mineralizing surface per bone surface (MS/BS), mineral apposition rate (MAR), and bone formation rate per bone surface (BFR/BS) were measured at the endosteal surface. (**E**) BFR/BS and MS/BS were measured at the trabecular surface. Bars indicate mean $\pm$ SD. The indicated *p*-values were calculated by unpaired *t*-test for equal or unequal variance (2E-BFR), or Rank sum test (if data is not normally distributed, 2A-Male, 2B-Female).

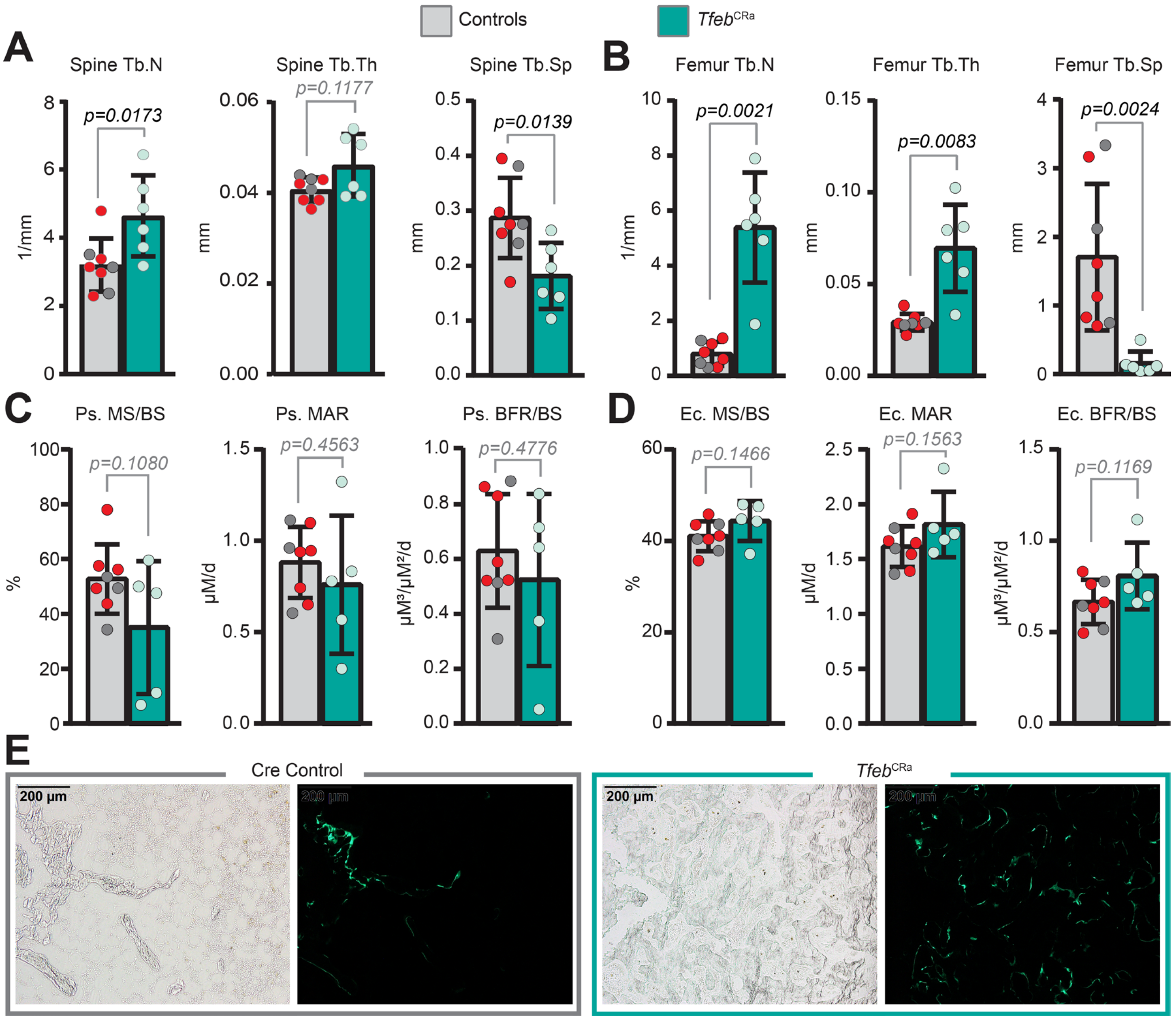

Supplemental Figure 3. *Tfeb* elevation is anabolic up to 12 months of age. (**A-B**) μCT analysis was performed on spine (L4) and femurs from 12-month-old female *Tfeb*^{CRa} mice and their littermate Cre controls (Osx1-Cre only [red dots)], Osx1-Cre;sgRNA$^{\textit{Tfeb}}$ [gray dots]). (**A- B**) Trabecular number (Tb.N), Trabecular thickness (Tb.Th), and trabecular spacing (Tb.Sp) measured in L4 (**A**) and at the femoral distal metaphysis (**B**). (**C-D**) Dynamic histomorphometry was performed on femurs of 12-month-old female *Tfeb*^{CRa} mice and their littermate Cre controls. MS/BS, MAR, and BFR/BS were measured at the periosteal (**C**) and endosteal (**D**) surfaces. (**E**) Light microscopy and fluorescent microscopy (GFP filter) images of femoral trabecular bone. n = 5-8 mice per group. Bars indicate mean $\pm$ SD. Indicated *p* values calculated by unpaired *t*-test for equal or unequal variance (3A-Tb.Th, 3B-Tb.N, 3B-Tb.Th), or Rank sum test (if data is not normally distributed, 3B-Tb.Sp).

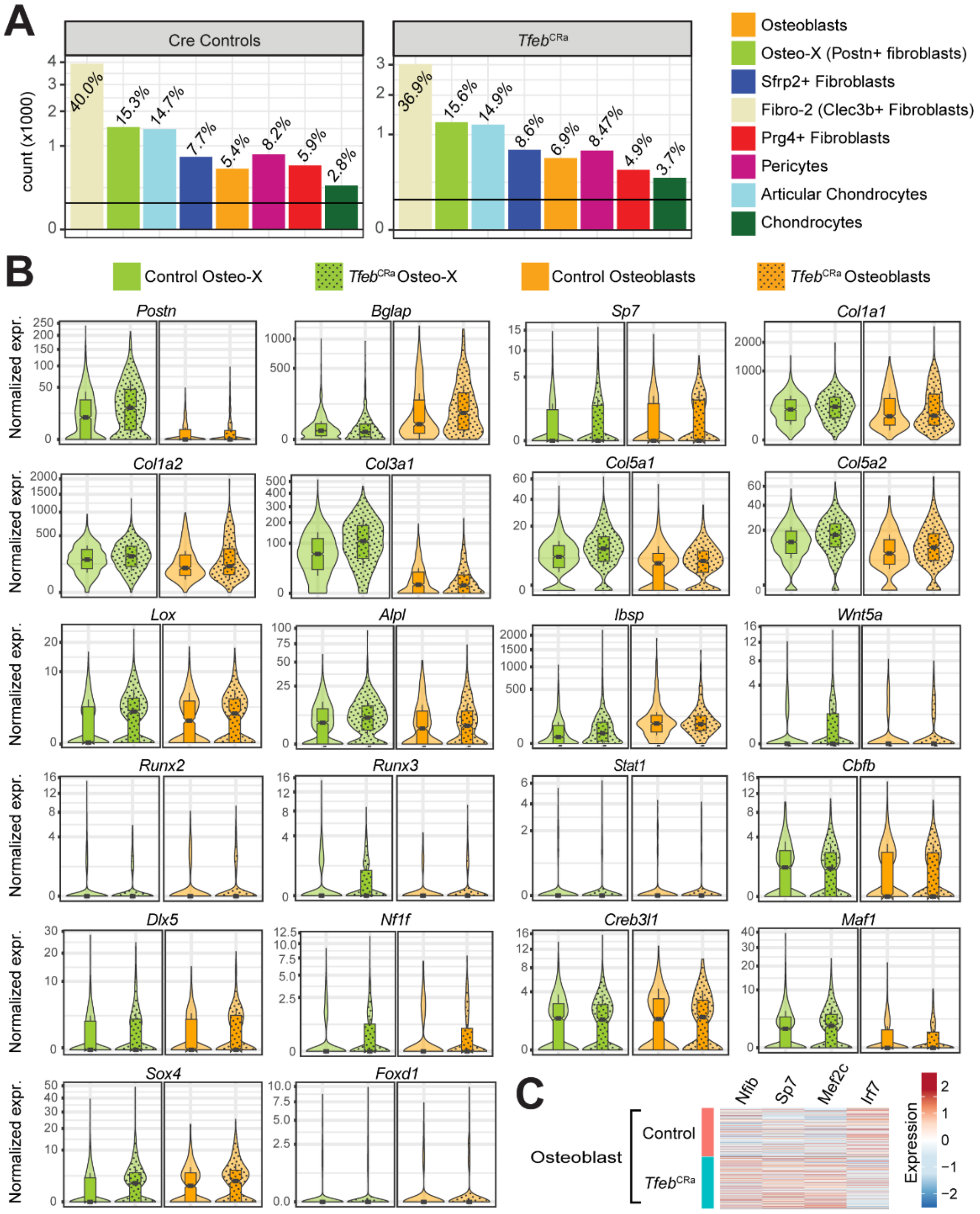

Supplemental Figure 4. *Tfeb* elevation increases expression of genes involved in osteoblast differentiation, bone matrix components, and bone matrix mineralization. Cells were obtained from femurs of 4-month-old female *Tfeb*^{CRa} mice and their Cre-expressing littermate controls (Osx1-Cre;CRa and Osx1-Cre;sgRNA^{Tfeb}), enriched in mesenchymal lineage cells, and subjected to single-cell RNA sequencing using the 10X Chromium platform. n = 2 mice per group. (**A**) Relative abundance of cells in each cluster. Exact percentages are indicated above each bar. (**B**) Violin plots of the expression of indicated genes in Osteo-X and Osteoblasts. (**C**) SCENIC analysis of transcription factor activity in Osteoblasts.

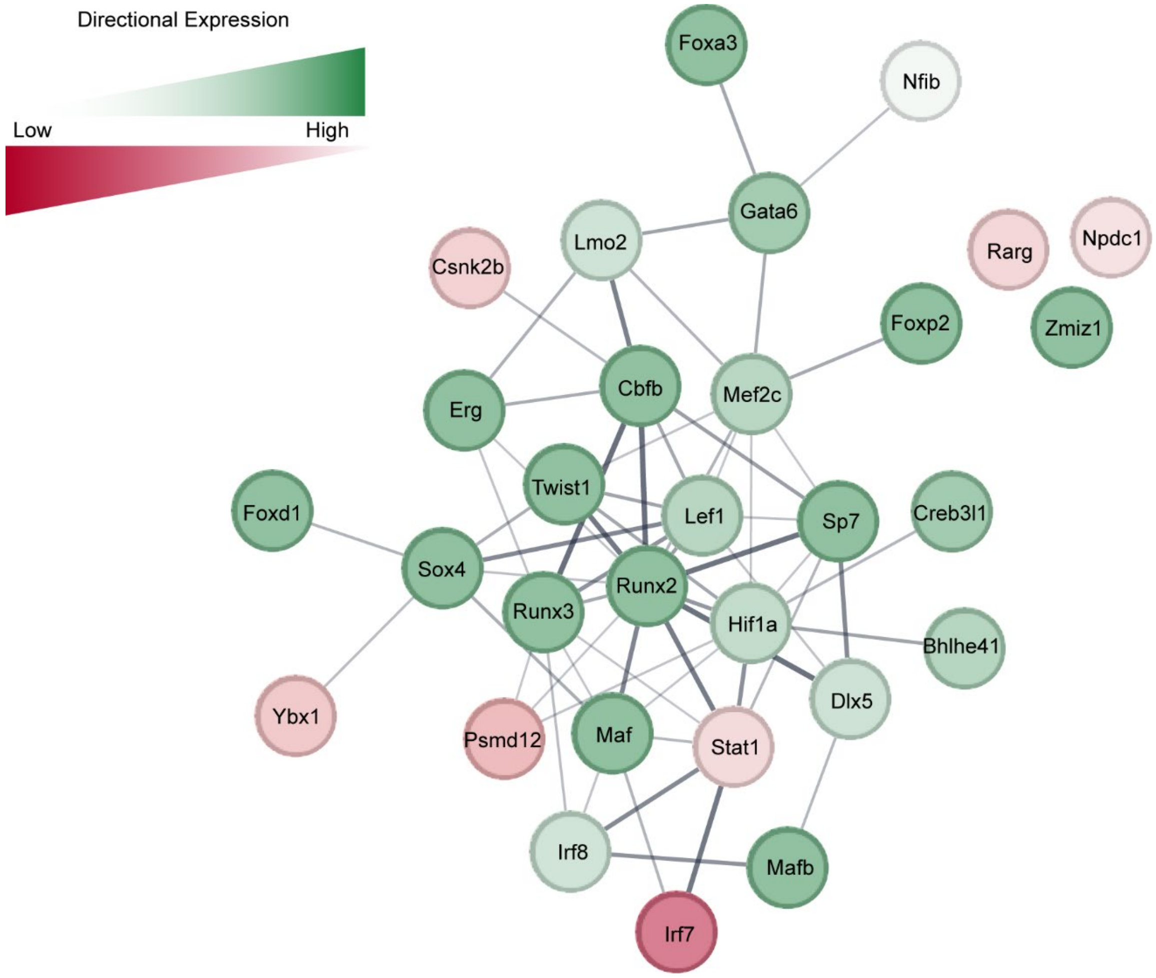

Supplemental Figure 5. *Tfeb*-induced changes in the transcriptional network of Osteo-X cells. Interactions of SCENIC identified transcription factors using STRING analysis. Increases (green) and decreases (red) in activity are indicated. Darker colors represent a higher magnitude of change. Lines indicate interaction between transcription factors, and the thickness of the lines indicates the strength of the interactions.

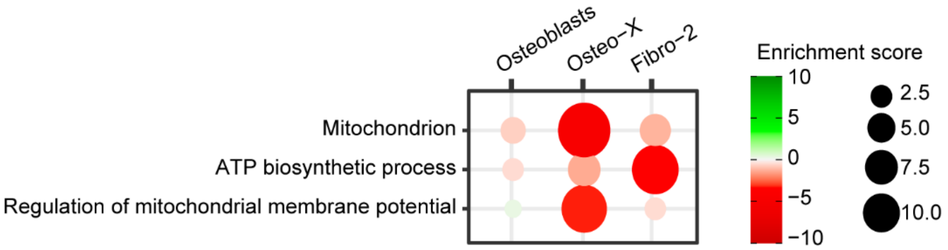

Supplemental Figure 6. Elevation of *Tfeb* decreases the expression of mitochondria-related genes. Gene Ontology (GO) analysis was performed using results of the scRNA-seq analysis of periosteal mesenchymal cells isolated from 4-month-old female *Tfeb*^{CRa} mice and their Cre-expressing littermate controls.
