# Supplementary figures and images for: Elevation of master autophagy regulator Tfeb in osteoblast lineage cells increases bone mass and strength
Source: JCI Insight. 2025 Jul 29;10(17):e191688. doi: 10.1172/jci.insight.191688 (PMC12487674; doi:10.1172/jci.insight.191688)

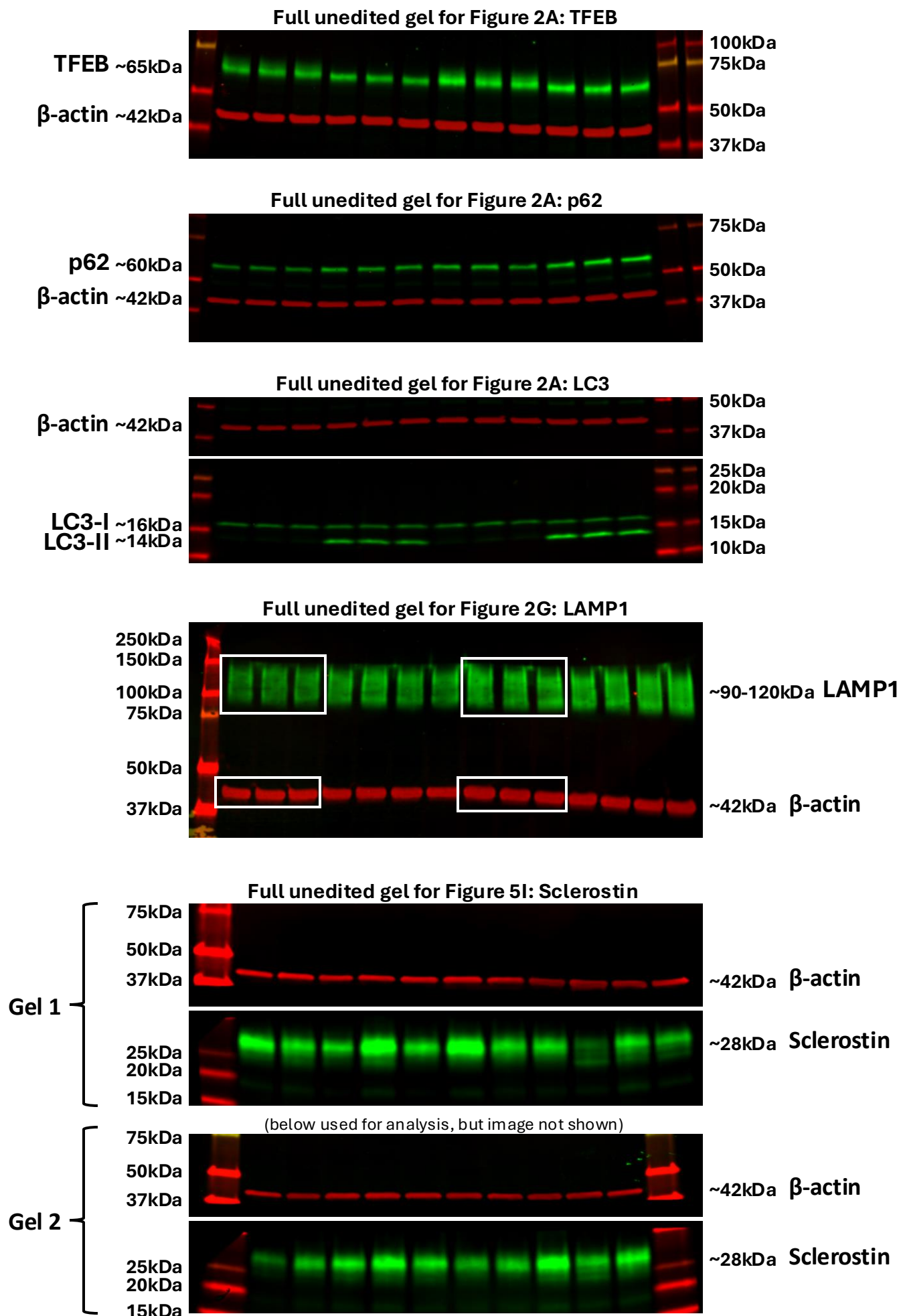

Supplement: Unedited blot and gel images [file jciinsight-10-191688-s249.pdf]
